# A review of the corrosion and wear resistance mechanisms of gas nitriding on steel

**DOI:** 10.1016/j.isci.2026.115048

**Published:** 2026-02-17

**Authors:** Junming Li, Hongyong Shao, Kai Lu

**Affiliations:** 1 School of Mechanical and Automotive Engineering, Guangxi University of Science and Technology, Liuzhou 545006, China

**Keywords:** Corrosion, Applied sciences, Engineering

## Abstract

This article presents a systematic evaluation of the mechanisms, recent process advances, and practical applications of gas nitriding for improving the corrosion and wear resistance of steels. It first revisits the thermodynamic and kinetic foundations of conventional gas nitriding, with particular emphasis on ammonia dissociation, nitrogen potential regulation, and the coupled processes of surface adsorption, dissolution, and diffusion. Subsequently, the microstructural characteristics of the compound layer and diffusion layer are summarized, and their respective roles in controlling frictional behavior and electrochemical performance are discussed. Emerging catalytic nitriding techniques, such as surface nanocrystallization (e.g., UNSM, SMAT, and SP), pre-oxidation-assisted gas nitriding, rare-earth or alloy-element catalysis, laser-assisted gas nitriding, and low-temperature nitriding, are reviewed. A comparative analysis highlights their effectiveness in improving hardness, wear resistance, and corrosion resistance. In addition, the synergistic mechanisms by which nitrided layers suppress micro-electrochemical heterogeneity, facilitate the formation of dense passivation films, and improve resistance to abrasive and adhesive wear through the dispersion of fine nitrides are analyzed. Finally, key scientific and engineering challenges are identified, particularly the absence of quantitative models describing corrosion-wear coupling at the microscale. In response to these gaps, prospective research directions are proposed, including multiscale mechanistic studies, high-fidelity simulations, scalable industrial routes for efficient low-temperature nitriding, and data-driven process-structure-property prediction frameworks enabled by machine learning. Collectively, this work establishes a theoretical foundation and research roadmap for optimizing nitriding processes and accelerating their industrial application in high-reliability engineering components.

## Graphical abstract

**Figure:**
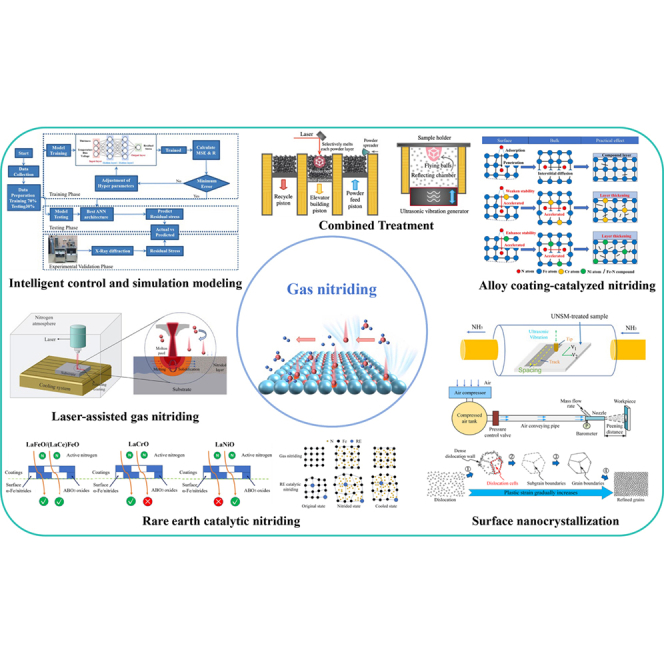

## Introduction

**Figure 1:**
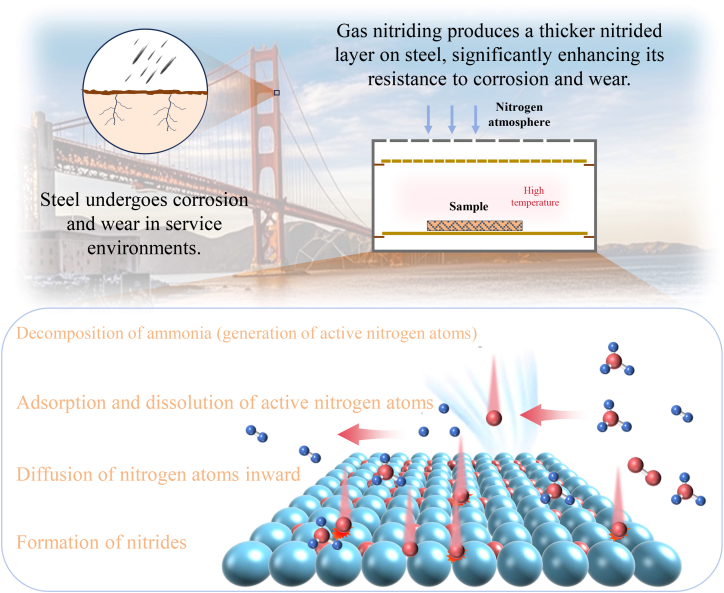
Schematic diagram of the gas nitriding mechanism

With the rapid development of industry, global demand for steel is showing a sustained growth trend. Steel, as a foundational material of modern industrial civilization, directly influences the service life, operational safety, and economic efficiency of mechanical equipment.^1^^,^^2^ However, in complex and demanding service environments (e.g., high temperature, high humidity, and high salt fog), steel surfaces are extremely susceptible to corrosion and wear.^3^ This not only leads to significant material loss and equipment maintenance costs but may also result in serious safety incidents.^4^^,^^5^ According to the Global Corrosion Survey Report, economic losses caused by steel corrosion account for approximately 3–4% of national GDP in surveyed countries. Moreover, energy and material losses due to wear mechanisms contribute to considerable resource wastage.^6^ Therefore, developing efficient, eco-friendly, and cost-effective surface protection technologies for steel to improve its corrosion and wear resistance, as well as overall performance, has become a critical research topic in materials science and surface engineering.^7^^,^^8^ Among the many surface-modification methods, gas nitriding (GN) is widely regarded as a key technique for enhancing the surface performance of steels due to its mature processing technology, broad applicability, and consistently strong strengthening effects.^9^ Compared with other surface-treatment techniques, GN offers several notable advantages. First, it produces a high-hardness, wear-resistant nitride layer on the surface—typically composed of γ′-Fe_4_N and ε-Fe_2-3_N—while preserving the toughness of the substrate. This layer markedly improves wear resistance and fatigue strength.^10^^,^^11^ Second, the process is compatible with a wide range of steel grades and components with complex geometries, giving it strong practical engineering value.^12^ In addition, unlike coating or thermal spraying methods, GN creates a metallurgically bonded nitride layer on the substrate, making it significantly more resistant to delamination.^13^ A schematic of the GN mechanism is shown in Figure 1. The nitriding layer significantly enhances the surface hardness, wear resistance, fatigue strength, and corrosion resistance of materials.^14^^,^^15^ However, traditional gas nitriding (TGN) suffers from issues such as slow diffusion rates, high energy consumption, and uneven layer thickness, limiting its industrial applications.^16^^,^^17^

To address these limitations, researchers are developing efficient catalytic nitriding technologies.^18^ The core of these technologies lies in modifying the thermodynamic and kinetic behavior of the nitriding process by introducing a catalytic medium, enabling low-temperature, high-speed, and high-quality nitriding.^19^^,^^20^ Current catalytic techniques primarily involve surface nanocrystallization (SNC) and surface-active catalysis nitriding.^21^ SNC creates nanocrystalline surface layers using high-energy plastic deformation techniques such as surface mechanical attrition treatment (SMAT), shot peening (SP), pre-oxidation (PO), and laser shock peening (LSP).^22^^,^^23^ The resulting grain boundaries provide high-density diffusion pathways for nitrogen atoms, significantly lowering the activation energy for diffusion.^24^^,^^25^ Surface-active catalysis activates the steel surface by introducing trace amounts of rare-earth (RE) or transition metal atoms (e.g., Ce, La, and Ni), catalyzing NH_3_ decomposition.^26^^,^^27^ Meanwhile, segregated RE atoms at grain boundaries accelerate nitrogen diffusion, refine nitride microstructures, and improve the densification of the nitrided layer.^28^ Additionally, computer-controlled GN utilizes real-time monitoring and feedback of nitrogen potential (K_N_) to precisely regulate the NH_3_ decomposition rate.^29^ This makes it possible to dynamically optimize the thickness, phase composition, and porosity of the nitrided layer, thereby meeting specific processing requirements.^30^

Based on the above discussion, this review analyzes the mechanisms of GN through process parameter optimization, surface nanocrystallization, surface activity catalysis, surface pre-oxidation, and surface laser treatment. The review evaluates the efficacy and limitations of these technologies in improving corrosion and wear resistance, while summarizing their industrial applications and future development trajectories. The future development of GN technology is moving toward more economic, environmentally friendly, efficient, and intelligent approaches. These innovations are expected to significantly enhance the corrosion and wear resistance of engineered steels, promoting sustainability in industrial production.

## Mechanisms of corrosion and wear in steel

Steel corrosion can be broadly classified into two fundamental categories according to the underlying mechanism: chemical corrosion and electrochemical corrosion. Chemical corrosion refers to material degradation resulting from the direct reaction of steel with dry gases or non-electrolytic media.^31^ In high-temperature environments, steel corrosion is predominantly chemical in nature and typically manifests as high-temperature oxidation, hydrogen embrittlement (HE), or sulfidation.^32^ When steel is heated below 570 °C, its reaction with O_2_ leads to the formation of oxide scales primarily composed of Fe_3_O_4_, with minor amounts of Fe_2_O_3_. At temperatures above 570 °C, FeO becomes the dominant oxide phase.^33^ In addition, CO_2_ and H_2_O vapor present in high-temperature atmospheres can further accelerate oxidation processes.^34^ Contact with hydrogen-containing compounds (e.g., HCl, H_2_S, and H_2_O) induces surface reactions that form FeCl_2_, FeS, and FeO, respectively, accompanied by the release of H_2_.^35^^,^^36^

Steel exposure to sulfur-containing environments at elevated temperatures results in sulfidation corrosion. This process is governed by the diffusion of sulfur species along surface defects and grain boundaries, where sulfide phases preferentially nucleate due to their higher chemical reactivity. As sulfidation proceeds, a sulfide scale develops and thickens through solid-state diffusion and interfacial reactions, leading to the progressive degradation of the load-bearing cross section and a marked reduction in the mechanical integrity of steel components.^37^

In most industrial environments, steel corrosion is dominated by electrochemical processes.^38^ Depending on the dominant cathodic reaction, electrochemical corrosion of steel can be categorized into hydrogen evolution corrosion and corrosion controlled by the oxygen reduction reaction (ORR). In strongly acidic environments, steel corrosion is accompanied by hydrogen evolution and is therefore referred to as hydrogen evolution corrosion.^39^ In this case, H^+^ ions in the surface electrolyte film accept electrons at the cathode and are reduced to H_2_. Simultaneously, anodic dissolution of iron produces Fe^2+^ ions, while the continuous dissociation of H_2_O leads to the accumulation of OH^−^ ions in the electrolyte. These OH^−^ ions react with Fe^2+^ to form Fe(OH)_2_, which can be further oxidized to Fe(OH)_3_ and subsequently dehydrated to form nFeO·mH_2_O, the principal constituent of rust.^40^

In weakly acidic or neutral environments, the oxygen reduction reaction predominates at the cathode, as dissolved O_2_ more readily accepts electrons than H^+^.^41^ The OH^−^ ions generated during ORR react with Fe^2+^ released at the anode to form Fe(OH)_2_, which is subsequently oxidized to Fe(OH)_3_ and ultimately transformed into rust. The chemical reactions for hydrogen evolution and oxygen corrosion of steel are as follows^42^^,^^43^:

Anodic oxidation:

(1) $$\text{Fe}\to {\text{Fe}}^{2+}+2e^{-}$$

Cathodic reduction (oxygen reduction reaction):

(2) $$O_{2}+2H_{2}O+4e^{-}\to 4{\text{OH}}^{-}$$

Cathodic reduction (hydrogen evolution corrosion):

(3) $$2H^{+}+2e^{-}\to H_{2}$$

Corrosion product formation:

(4) $${\text{Fe}}^{2+}+2{\text{OH}}^{-}\to \text{Fe}{\left(\text{OH}\right)}_{2}$$

(5) $$4\text{Fe}{\left(\text{OH}\right)}_{2}+O_{2}+2H_{2}O\to 4\text{Fe}{\left(\text{OH}\right)}_{3}$$

(6) $$4{\text{Fe}}^{2+}+O_{2}+\left(4+x\right)H_{2}O\to 2{\text{Fe}}_{2}O_{3}+xH_{2}O$$

**Figure 2:**
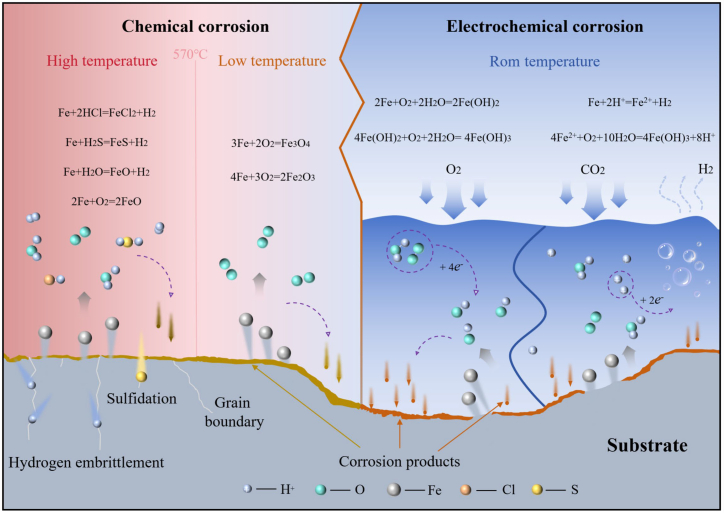
Schematic diagram of the corrosion mechanism of steel

A schematic illustration of the steel corrosion mechanism is shown in Figure 2. A thorough understanding of these mechanisms and their governing conditions is essential for the effective prevention and control of steel corrosion. In practical industrial applications, corrosion behavior is often strongly coupled with wear, giving rise to corrosive wear phenomena. Electrochemical corrosion alters the surface chemistry and microstructure of steel, resulting in the formation of corrosion products that are generally porous and mechanically weak, thereby reducing surface hardness and load-bearing capacity. Mechanical wear, in turn, removes protective oxide or corrosion product layers, continuously exposing fresh metal surfaces to the corrosive environment and accelerating electrochemical reactions.^37^ This synergistic interaction between electrochemical corrosion and mechanical wear significantly increases material degradation rates and must be considered when designing strategies to mitigate steel failure and extend component service life.

### Wear mechanisms

**Figure 3:**
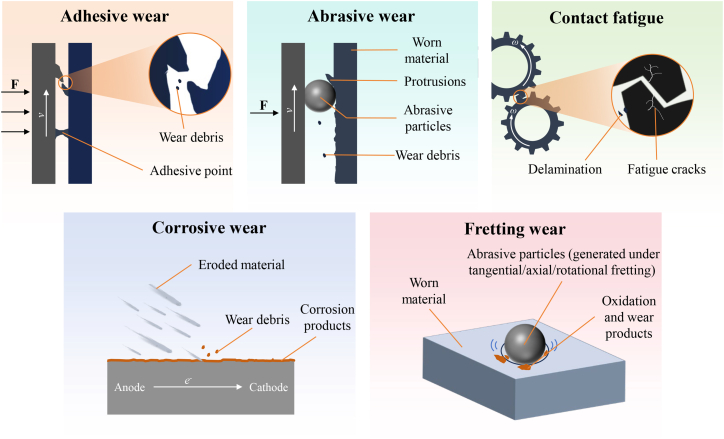
Predominant wear modes of steel

From a tribological perspective, wear of steel can be classified into several fundamental types according to the prevailing damage mechanism, namely adhesive wear, abrasive wear, contact fatigue, corrosive wear, and fretting wear.^44^ Schematic illustrations of these mechanisms are provided in Figure 3. Adhesive wear occurs when two metallic surfaces, or a metal surface and its counterface, are brought into contact under load and undergo relative motion. At the microscopic scale, surface asperities experience high local stresses, leading to plastic deformation and the formation of junctions or micro-welds due to strong interatomic interactions. During sliding, these junctions are sheared, resulting in material transfer or detachment and the generation of wear debris.^45^ Adhesive wear is particularly severe under conditions of poor lubrication or high contact stress.

Abrasive wear arises when hard asperities or entrained hard particles slide against a relatively softer steel surface. Depending on the contact geometry and hardness ratio, the dominant material removal mechanisms include micro-cutting and plowing.^46^ Abrasive wear typically manifests as parallel grooves or scratches on the worn surface and is prevalent in environments containing hard contaminants, such as mineral particles or oxides.^47^

Contact fatigue is associated with cyclic or repeated contact stresses, such as those encountered in rolling bearings, gears, and rails. Under cyclic loading, stress concentrations develop at or beneath the surface, leading to the initiation of microcracks.^48^ With continued loading, these cracks propagate and coalesce, ultimately resulting in surface spalling or flaking. Unlike adhesive or abrasive wear, contact fatigue is primarily governed by stress amplitude, contact geometry, and material microstructure rather than by sliding distance alone.^49^

In addition to purely mechanical wear, steel is susceptible to various forms of corrosion, which are electrochemical in nature. Uniform corrosion involves relatively even metal dissolution over the exposed surface, whereas localized corrosion, such as pitting or crevice corrosion, leads to highly concentrated material loss in confined regions. These corrosion processes can significantly alter surface chemistry and mechanical properties, thereby influencing subsequent wear behavior.^50^

Corrosive wear, also referred to as tribocorrosion, represents the coupled interaction between electrochemical corrosion and mechanical wear. In corrosive environments, electrochemical reactions lead to metal dissolution and the formation of corrosion products or passive films.^51^ Mechanical action, such as sliding or abrasion, repeatedly removes these surface films, exposing fresh metal to the environment and accelerating electrochemical reactions.^52^ Simultaneously, corrosion degrades the surface integrity of steel, reducing hardness and facilitating crack initiation, which increases its susceptibility to mechanical wear. As a result, the total material loss under corrosive wear conditions is greater than the sum of corrosion and wear acting independently.^53^

Fretting wear occurs under conditions of small-amplitude oscillatory motion between contacting steel surfaces, typically induced by vibration. The repeated micro-slip leads to severe stress concentration, surface oxidation, and the generation of fine debris, which often exacerbates damage through secondary abrasive action.^54^

In summary, the corrosion and wear behavior of steel is governed by multiple, often interacting mechanisms. A clear distinction between individual wear and corrosion types, as well as an understanding of their synergistic effects, is crucial for the rational design of steels and surface treatments intended for demanding service environments.

### Corrosion and wear-resistant technologies for steel

Although corrosion and wear of steel are inevitable during service, strategies such as matrix alloy design, surface-enhancement techniques, and lubrication modification can significantly extend equipment service life.^55^ Among these, surface-enhancement techniques have emerged as a core approach for preventing steel corrosion and wear due to their process flexibility and cost-effectiveness. Commonly used surface-enhancement technologies today include thermochemical treatment, thermal spraying, and laser cladding.^56^^,^^57^ Their key characteristics are summarized later in discussion.

**Figure 4:**
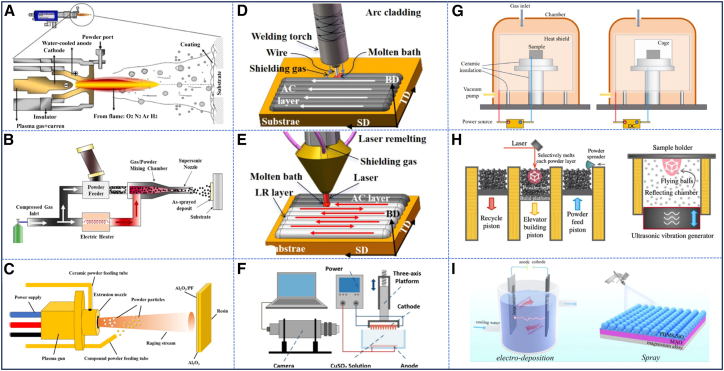
Schematic diagrams of various surface treatment technologies (A–I) Are schematic diagrams illustrate the mechanisms of thermal spraying (reprinted from Lv et al.^58^ Copyright 2024, with permission from Elsevier), cold spraying (reprinted from Mengiste et al.^59^ Copyright 2025, with permission from Elsevier), plasma spraying (reprinted from Chen et al.^60^ Copyright 2025, with permission from Elsevier), arc cladding, laser cladding (reprinted from Li et al.^59^ Copyright 2025, with permission from Elsevier), electrochemical deposition (reprinted from Xu et al.^61^ Copyright 2024, with permission from Elsevier), plasma nitriding, surface mechanical alloying,^62^ and superhydrophobic surface coating technologies (reprinted from Song et al.^63^ Copyright 2024, with permission from Elsevier)., respectively.

Thermochemical treatment heats the component in a reactive medium so that one or more elements (such as C, N, or B) diffuse into the surface layer, altering its composition and microstructure. Thermal spraying uses a heat source—such as an electric arc, plasma, or flame—to melt or semi-melt the coating material. The molten droplets are then atomized and propelled onto the substrate by a high-velocity gas stream to form a layered coating. Cold spraying accelerates solid particles to supersonic velocities using preheated high-pressure gas. The particles undergo intense plastic deformation upon impact, enabling them to bond and form a coating. Laser cladding uses a high-energy laser beam to create a molten pool on the substrate surface. Powder or wire feedstock is injected into this pool, where it melts together with a thin layer of the substrate and rapidly solidifies to form a dense, metallurgically bonded coating. Arc cladding employs an electric arc to melt a filler wire, which is then deposited on the substrate to form a relatively thick coating. Electroless plating uses a reducing agent in solution to deposit metal or alloy coatings through an autocatalytic reaction on the catalytically active substrate surface. Electrochemical deposition applies an external electric field that drives metal cations to the cathode (the workpiece), where they are reduced and deposited as a coating. Surface mechanical alloying uses high-energy ball milling or shot peening to induce severe plastic deformation at room temperature, incorporating second-phase particles and generating nonequilibrium structures such as amorphous layers, nanocrystalline surfaces, or supersaturated solid solutions. The mechanisms of these techniques are illustrated in Figure 4. In addition, Table 1 summarizes the advantages and limitations of each method in terms of process applicability, coating performance, and environmental impact. It is important to note that evaluating these advantages and drawbacks involves balancing multiple objectives. No single technique is universally superior; its suitability depends entirely on the specific application and performance requirements.

**Table 1:** Advantages and disadvantages of different surface treatment technologies

Type	Advantage	Disadvantage	Reference
Chemical Heat Treatment	High interfacial bonding strength, no thickness limitation, minimal substrate distortion.	Long processing cycles, limited to diffusible elements, and geometrical constraints on complex shapes.	Luo et al.^64^
Thermal Spraying	Broad material compatibility, thick-coating capability (>5 mm), and field applicability.	High porosity (5–15%), low bond strength (mechanical anchoring), and significant thermal stress induction.	Lv et al.^58^
Laser Cladding	Metallurgical bonding, precise controllability, and low dilution rate (<5%).	High capital cost, limited deposition rate (<1 kg/h), and line-of-sight geometric restrictions.	Yongcun et al.^59^
Cold Spraying	Low-temperature process (<150°C), retention of nanostructured feedstock, high deposition efficiency.	Difficulty spraying brittle materials, inherent coating porosity (1–3%), and high process gas consumption.	Mengiste et al.^65^
Plasma Spraying	High-temperature capability, high coating purity, and excellent thickness uniformity (±10%).	Complex equipment requirements, high residual tensile stress, and low powder utilization rate (40–60%).	Chen et al.^60^
Arc cladding	Lowest operational cost, high deposition rate (>20 kg/h), and exceptional portability.	Severe in-flight oxidation, weak bond strength (15–30 MPa), and high surface roughness (Ra 20–50 μm).	Liu et al.^66^
Electroless Plating	No external current requirement, uniform coverage on complex geometries (±5% thickness deviation).	Low deposition rate (5–20 μm/h), toxic wastewater (Ni^2+^/hypophosphite), high chemical reagent cost.	Geng et al.^67^
Electrochemical Deposition	Zero thermal distortion risk, precision thickness control (±0.1 μm), and high electrical conductivity.	Hydrogen embrittlement risk, edge effect, and environmental compliance challenges.	Xu et al.^61^
Surface Mechanical Alloying	Synthesis of immiscible material systems (e.g., Al-Pb), contamination-free processing, nanoscale grain refinement (10–50 nm).	Contamination from milling media, extremely high energy consumption (>5,000 kWh/ton), and limited scalability.	Chen et al.^68^
Superhydrophobic Surface Coating	Excellent self-cleaning properties and outstanding corrosion resistance.	Limited durability, poor chemical stability, and high-cost implication.	Song et al.^63^

A comprehensive analysis shows that thermochemical treatments are highly effective in achieving coordinated strengthening of both the substrate and the surface layer by precisely adjusting the chemical composition and microstructure of the material’s surface.^69^ This technology not only gives steel excellent wear resistance and good corrosion resistance, but also has become the mainstream surface-enhancement technology due to its simple process and low cost.^70^ Currently, the most widely applied chemical heat treatment techniques primarily include carburizing, boriding, and nitriding. Among these, carburizing is typically conducted within the temperature range of 850°C–950°C. Hydrocarbon gases such as CH_4_ and C_3_H_8_ decompose at elevated temperatures during this process, releasing active carbon atoms and then diffusing into steel surfaces. Ultimately, this process forms a strengthened layer consisting of high-hardness carbides or high-carbon martensite, typically achieving case depths of 0.8–2.0 mm and exhibiting outstanding crushing resistance.^71^ Qin et al.^72^ investigated the effect of carburizing treatment on the rotating bending fatigue performance of 18CrNiMo7-6 steel. They found that the fatigue limit of as-received (AR) samples was 680 MPa, whereas carburized samples achieved a fatigue limit as high as 1108 MPa, representing a 62.9% increase. Additionally, Wang et al.^71^ developed a hard carburized layer exceeding 1400 μm in thickness on DIEVAR iron-based alloy using solid-state carburization. The hardness value of the carburized layer decreases as the carbon concentration decreases, and the surface hardness is 272% higher than that of the non-carburized test piece. Shi et al.^73^ further reported enhanced surface properties of steel through cyclic carburizing. The results showed that the number of cyclic carburization cycles had a significant effect on tribological behavior, and that the hardness of the steel was positively correlated with the number of cyclic carburization cycles.

Boriding is typically performed at 850°C–1000°C, commonly employing boron-containing agents such as borax, ferroboron alloy, or BCl_3_ as diffusants.^74^ During this process, active boron atoms diffuse into the substrate, forming a surface layer composed of high-hardness borides. This layer exhibits extremely high microhardness and exceptional wear resistance, rendering it particularly suitable for environments with severe wear conditions.^75^ However, the hardened layer exhibits relatively limited thickness and slightly higher brittleness compared to other surface treatments.^76^ Panda et al.^77^ investigated the effect of boronizing on the tribological properties of 316L stainless steel. The results indicated that the multilayered structure of surface compounds formed after boronizing reduced crack propagation capability, leading to a 20%–40% reduction in the coefficient of friction (COF). Medvedovski et al.^78^ further documented field testing of boronized steel in geothermal power plants. The study revealed that the chemical inertness of crystalline iron borides and their compact dual-layer structure actively suppressed the initiation and propagation of surface microcracks. While boriding treatment significantly enhances the wear resistance of steel substrates, recent findings indicate that this process paradoxically reduces their corrosion resistance. Çetin et al.^79^ investigated the wear and corrosion characteristics of borided AISI 904L steel. Wear tests demonstrated a 40-fold improvement in the wear resistance of the borided steel compared to untreated material. However, its corrosion resistance in a 3.5 wt % NaCl solution was found to be lower than that of the AR samples.

In contrast, nitriding is typically carried out at lower temperatures (420°C–580°C) by decomposing NH_3_ to provide [N] to the steel.^80^ N atoms diffuse into the surface of the steel to form a hard nitride (such as ε-Fe_3_N and γ′-Fe_4_N) strengthening layer.^81^ Although its hardness is slightly lower than that of borided layers, it exhibits excellent wear resistance, fatigue resistance, and exceptional corrosion resistance.^82^ Dalibón et al.^83^ subjected AISI 420 martensitic stainless steel to short-duration nitriding treatments. Their study revealed that samples nitrided at 440°C exhibited optimal wear and corrosion resistance, a behavior attributed to the presence of nitrides. Building on this foundation, Ostrovski et al.^84^ combined laser surface melting with nitriding technology. Experimental results demonstrated that this hybrid process significantly increased surface hardness to three times that of the substrate while effectively reducing the material’s mean wear rate. Huang et al.^85^ further investigated the elevated-temperature wear behavior of nitrided die steel. The results showed that the wear rate of the nitrided samples at room temperature was reduced by 92.6%, and the wear mechanism changed from severe adhesive wear and fatigue wear to mild abrasive wear.

Table 2 summarizes the corrosion resistance of various steels after chemical heat treatment, where I_corr_ (corrosion current density) and E_corr_ (corrosion potential) serve as fundamental parameters for the quantitative assessment of corrosion behavior.^86^ I_corr_ reflects the corrosion rate of a material, with lower values indicating a slower corrosion process. E_corr_ reflects the tendency of a material to corrode, and a positive shift indicates that the material is more likely to become passivated.^83^ It can be observed that samples subjected to nitriding treatment universally exhibit lower I_corr_ compared to those processed by carburizing or boriding. This indicates that nitriding technology delivers the most significant enhancement to the corrosion resistance of steel substrates.

**Table 2:** Corrosion resistance comparison of steel substrates after chemical heat treatment

Substrates	Diffusion elements	Corrosive medium solution	Electrochemical corrosion results		Reference
–	–	–	E_corr_(mV)	I_corr_(μA/cm^2^)	–
316LVM steel	C	3.5 wt % NaCl	−189	0.672	Juri et al.^26^
18CrNiMo7-6 steel	C	3.5 wt % NaCl	−563.1	7.511	Wang et al.^87^
20MnCr5 steel	C	1.04wt%NaHSO_3_	−645	4.87	He et al.^88^
AISI 321 steel	C	3.5 wt % NaCl	−513	1.5	Savrai et al.^89^
AISI H13 steel	C	3.5 wt % NaCl	−656	0.875	Lotfi-Khojasteh et al.^90^
AISI 904L steel	B	3.5 wt % NaCl	−317	0.45	Çetin et al.^79^
AISI H13 steel	B	3.5 wt % NaCl	−560	7.499	Kayalı et al.^91^
High manganese steel	B	3.5 wt % NaCl	−673.5	4.75	Sezgin et al.^92^
R4 grade steel	B	3.5 wt % NaCl	−633	1.98	Alkan et al.^93^
AISI 430 FSS steel	B	0.27M KCl	−132.4	0.01	Silva et al.^94^
316L steel	N	3.5 wt % NaCl	−187	0.31	Godec et al.^95^
P20 steel	N	3.5 wt % NaCl	−538.3	3.7	Yan et al.^17^
AISI H13 steel	N	Natural seawater	−497	0.143	Fernandes et al.^96^
UNS S31254	N	3.5 wt % NaCl	−108	0.19	Kurelo et al.^97^
300M steel	N	5.0 wt % NaCl	−110	0.03	Zhao et al.^98^

Nitriding technology can be categorized into plasma nitriding (PN), salt bath nitriding, and GN.^99^ PN is conducted within a vacuum chamber where the workpiece functions as the cathode and the furnace wall serves as the anode. A nitrogen-containing gas is introduced into the vacuum environment, where glow discharge plasma is generated through the application of a high-voltage electric field.^100^ High-energy nitrogen ions bombard the workpiece surface, elevating its temperature to the nitriding range while simultaneously enabling the diffusion of [N] into the material subsurface.^101^ PN offers high process efficiency and enables precise control over critical parameters, including temperature, gas pressure, and applied voltage, thereby achieving the accurate regulation of case layer composition and thickness.^102^ However, PN is highly efficient and can precisely control process parameters such as temperature, pressure, and voltage, enabling precise control of the composition and thickness of the diffusion layer.^99^ However, PN is greatly affected by the geometric shape of the workpiece. For example, deep holes and narrow gaps in workpieces can easily cause fluctuations in the thickness of the diffusion layer due to uneven glow, and the high equipment investment cost makes it difficult to promote on a large scale.^103^ Salt bath nitriding is performed in molten salt baths, primarily categorized into low-temperature type (approximately 520°C) and high-temperature type (570°C–580°C).^104^ The Quench-Polish-Quench duplex treatment is the predominant technique, comprising multiple processes including nitriding, oxidizing, polishing, and re-oxidizing.^105^ Due to the high thermal conductivity of the molten salt in salt bath nitriding, the temperature is uniformly distributed across all parts of the workpiece, making it particularly suitable for nitriding complex geometric components. However, the most significant drawback of salt bath nitriding is the substantial environmental pollution, as the cyanide-containing salt baths require rigorous wastewater treatment.^106^ GN involves introducing NH_3_ into a sealed furnace chamber, where, at temperatures ranging from 500°C to 570°C, [N] are generated through the decomposition of NH_3_ and subsequently diffuse into the surface layer of steel components.^107^ The GN process is well-developed and mature, making it highly suitable for surface nitriding of large workpieces and components with complex geometries. Moreover, the nitrides formed in the surface layer are denser, resulting in superior corrosion resistance.^108^

Comprehensive analysis reveals that GN exhibits distinctive overall advantages among the existing surface-enhancement techniques for steels. This technique not only offers relatively lower processing costs and favorable process controllability but also significantly enhances the corrosion and wear resistance of the treated steel surface.^109^ Therefore, from the perspectives of technical economy, process applicability, and performance enhancement, GN is undoubtedly the best choice for solving the problem of steel corrosion and wear.

## Gas nitriding mechanisms and the wear and corrosion behavior of nitrided layers

This chapter provides a systematic overview of the fundamental principles and kinetic processes of GN, as well as the key properties of the nitrided layers formed on steel surfaces. The focus is on elucidating the wear and corrosion mechanisms of nitrided layers and comparing how nitriding conditions, microstructure, and performance are interrelated across different material systems. These analyses offer a theoretical basis for targeted process optimization.

### Mechanisms of gas nitriding and material-specific considerations

**Figure 5:**
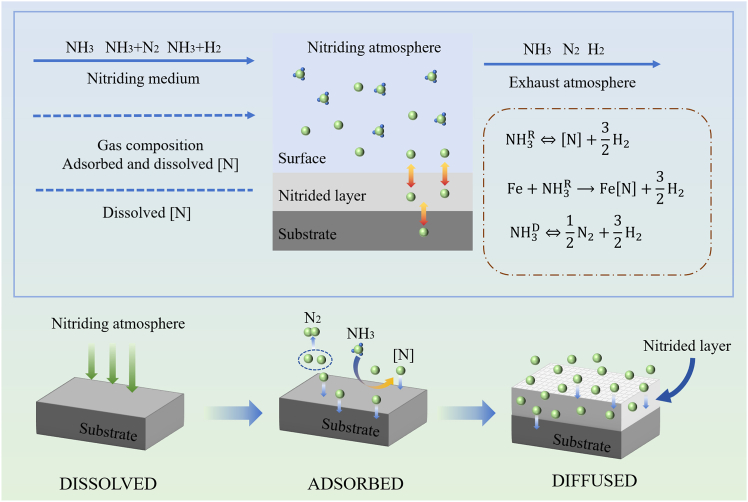
The reaction process of gas nitriding

As a major surface-strengthening technique, GN is grounded in thermodynamics, kinetics, and materials science. During the process, the component is exposed to a nitrogen-rich atmosphere at elevated temperatures.^110^ Nitrogen atoms adsorb onto the metal surface, dissolve into the lattice, and diffuse inward, ultimately forming a high-performance nitrided layer. A schematic of the GN reaction mechanism is shown in Figure 5. When ammonia is introduced into the furnace, it decomposes on catalytic surfaces as follows^111^:

(7) $${\text{NH}}_{3}^{R}⇄\left[N\right]+\frac{3}{2}H_{2}$$

(8) $$\text{Fe}+{\text{NH}}_{3}^{R}\to \text{Fe}\left[N\right]+\frac{3}{2}H_{2}$$

This reaction is reversible. When it proceeds to the right, active nitrogen [N] is produced; when it shifts to the left, [N] recombines to form N_2_, which then escapes from the system.^112^ The nitriding process requires a continuous supply of NH_3_ and careful control of its dissociation rate to maintain a sufficient K_N_ in the furnace, thereby driving the reaction toward the formation of active nitrogen. The dissociated [N] is highly reactive and serves as the primary species that diffuses into the steel substrate. K_N_ is a key parameter in modeling the growth kinetics of the nitrided layer.^113^

(9) $$K_{N}=\frac{P_{NH_{3}}}{{\left(P_{H_{2}}\right)}^{1.5}}$$

Here, $P_{NH_{3}}$ represents the partial pressure of NH_3_ in the exhaust gas, and $P_{H_{2}}$ represents the partial pressure of H_2_. The flow rate of NH_3_ directly affects its dissociation rate and is the primary means of regulating the K_N_ at the workpiece surface.^114^ The mathematical relationship between K_N_ and the NH_3_ dissociation rate *ω* is defined as follows:

(10) $$K_{N}=\frac{1-\omega }{{\left(0.75\omega \right)}^{1.5}}$$

*ω* represents the fraction of ammonia that decomposes during nitriding relative to the total amount of ammonia introduced. K_N_ and *ω* exhibit a strong negative correlation. Physically, ω reflects the extent of ammonia decomposition, whereas K_N_ represents the nitriding driving force imposed by the furnace atmosphere under specific process conditions.^115^

**Figure 6:**
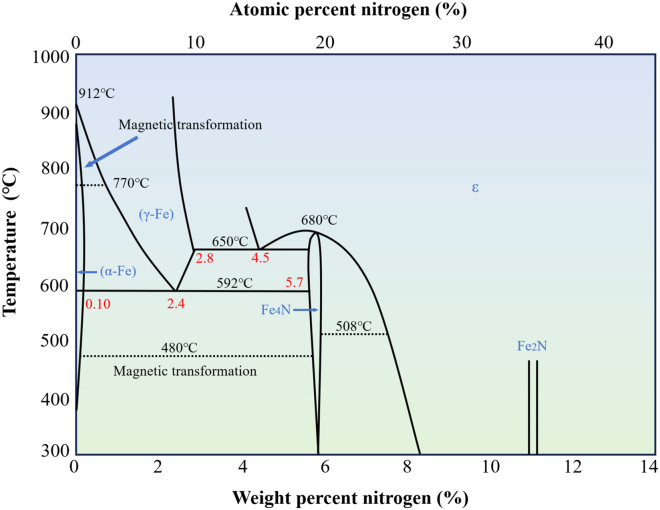
Fe-N equilibrium phase diagram

GN is fundamentally a complex physicochemical process involving gas-phase decomposition, surface adsorption, and subsurface diffusion. From a thermodynamic perspective, the key is controlling K_N_, which defines the atmosphere’s capacity to supply active nitrogen species.^116^ By regulating the ammonia dissociation rate and temperature, the phase constitution of the compound layer formed on the steel surface can be precisely controlled. The Fe-N equilibrium phase diagram (Figure 6) provides a clear process window for optimizing phase evolution during nitriding.^117^ For alloy steels, advanced thermodynamic calculations (CALPHAD) can predict the formation of stable nitrides by elements such as Cr and Al, thereby modifying phase equilibria. From a kinetic standpoint, the nitriding process is primarily governed by the diffusion rate of nitrogen atoms within the ferritic matrix. Nitrogen diffusion in the diffusion zone can be described by Fick’s second law^118^:

(11) $$\frac{\partial C\left(x,t\right)}{\partial t}=D_{N}\frac{\partial^{2}C\left(x,t\right)}{\partial x^{2}}$$

Where *C(x, t)* is the nitrogen concentration at a distance *x* from the surface at time *t*. *D*_*N*_ is the diffusion coefficient of nitrogen in ferrite, which strongly depends on temperature and follows the Arrhenius relationship:

(12) $$D_{N}=D_{0}\;\exp\left(-Q/RT\right)$$

Where *Q* is the activation energy for diffusion, a higher *Q* value corresponds to a greater sensitivity of the diffusion rate to temperature. *T* is the absolute temperature, and *R* is the universal gas constant (≈8.314 J mol^−1^ K^−1^),^119^ which ensures dimensional consistency within the equation. It ensures the units on both sides of the equation are consistent. Therefore, under ideal conditions with a constant surface concentration, the relationship between case depth *d* and time *t* is approximately given by:

(13) $$d\propto \sqrt{D_{N}\cdot t}$$

The growth of the compound layer also follows a parabolic law. Modern computational simulations play a crucial role in overcoming the limitations of classical theory. First-principles calculations reveal the dissolution and migration barriers of nitrogen in iron at the atomic scale. Kinetic simulations based on thermodynamic and diffusion databases (e.g., DICTRA) accurately predict the nitrogen concentration profile and case depth under various processing conditions. This enables “virtual experiments” and significantly reduces experimental trial-and-error.^120^

In summary, integrating thermodynamic equilibrium analysis, kinetic diffusion theory, and advanced computational simulations enables a comprehensive understanding and optimization of the GN process across scales, from atomic to macroscopic. This multi-scale approach enables precise control of surface phase composition and case depth, and promotes the transition of GN technology from experience-based “craftsmanship” to predictable, designable “science.” It establishes a solid foundation for intelligent process control and new material development.

### Microstructural features of the nitrided layer

The microstructure and composition of the nitrided layer are mainly influenced by nitriding temperature, K_N_, processing time, and cooling method. Table 3 summarizes the phases and their characteristics in the Fe-N system. The typical microstructure composition of the nitrided layer from the surface inward is as follows.

**Table 3:** Phases and their characteristics in the Fe-N system

Phase	Symbol	Crystal structure	Nitrogen atom location	Nitrogen solubility (at.N%)
Nitrogen-bearing ferrite	α	BCC	Octahedral interstices	0–0.4
Nitrogen-bearing austenite	γ	FCC	Octahedral interstices	0–10.8
Nitrogen-bearing martensite	α′	BCC	Face center of the unit cell	0.7–10.8
		BCT	Edge center	2.7–10.8
Fe_4_N phase	γ′	FCC	Body center	19.3–20
Fe_2-3_N phase	ε	HCP	Octahedral interstices	15–33
Fe_2_N phase	ζ	Orthorhombic lattice	Rhombic dodecahedral interstices	0–33.2
Nitrogen metastable phase	α''	Pseudo-FCC	Octahedral interstices	11.1

#### Outermost layer: high nitrogen compound layer (ζ phase or ε phase)

ζ phase: This phase only appears at extremely high nitrogen concentrations and nitriding temperatures below 500°C. Due to its narrow formation conditions, it rarely appears in practical nitriding processes.^121^ ε phase: This is the main surface compound phase, with a wide nitrogen concentration range. It is stable within the typical nitriding temperature range of 500°C–590°C. A notable characteristic of these phases is the hexagonal close-packed (HCP) structure, where nitrogen atoms occupy interstitial sites in an ordered arrangement.^122^ These phases are hard and brittle, providing primary wear resistance. However, overly thick layers are prone to spalling.

#### Subsurface layer: transition zone between ε phase and γ′ phase

The subsurface primarily includes the transition region where the ε phase transforms into the γ′ phase. From the surface inward, the nitrogen concentration gradually decreases.^123^ When nitrogen concentration drops to about 5.75 wt %, the ε phase transforms into the γ′ phase. The nitrogen concentration in this subsurface layer lies between the solubility lower limit of ε phase and the solubility upper limit of γ′ phase. It retains high hardness, but brittleness is slightly lower than that of the pure ε phase layer. The γ' phase has a face-centered cubic (FCC) structure, with nitrogen atoms occupying body-centered positions in an ordered manner.^124^

#### Diffusion layer (main body): γ′ phase and α phase (or nitrogen-rich martensite α')

The diffusion layer is characterized by a further decrease in nitrogen concentration and is the thickest part of the nitrided layer. Its final microstructural form is closely related to cooling speed.^125^ When nitrogen concentration is relatively high and cooling speed is slow, the γ′ phase region is preserved during cooling. When the nitriding temperature exceeds 590°C (leading to γ phase formation), and subsequent cooling is slow, the γ phase region undergoes a eutectoid transformation, causing γ phase to decompose into α phase and γ' phase.^126^ This results in a mixture of α ferrite and γ′ phase exhibiting lamellar or granular morphology. However, if the cooling rate is too fast, the γ to α and γ' phase transformation is suppressed, leading to a diffusionless martensitic transformation from γ phase to α′ phase. This high-hardness, high-strength supersaturated solid solution forms a needle-like or plate-like martensitic structure with hardness higher than the eutectoid structure obtained by slow cooling in the same alloy.^127^

**Figure 7:**
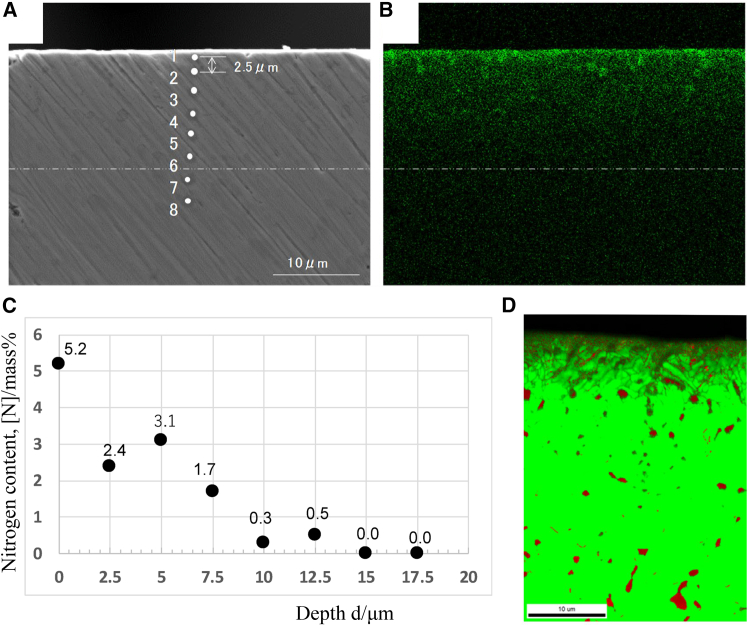
SEM image and nitrogen distribution map on the cross-section of AISI420 nitrided at 673 K for 3.6 ks (A) SEM image. (B) Nitrogen distribution map obtained by EDX. (C) Nitrogen depth profile of the nitrided specimen. (D) Phase map on the cross-section of the nitrided specimen.^128^

Figure 7 illustrates the correlation between nitrogen diffusion behavior and the resulting microstructural and phase evolution in AISI 420 steel nitrided at 673 K for 3.6 ks. Figure 7A shows a cross-sectional SEM image of the nitrided layer, revealing a distinct near-surface modified zone with a thickness of approximately 10 μm. This region exhibits a noticeably refined microstructure compared with the substrate, indicating significant nitrogen incorporation and associated phase transformation. The dotted line marks the approximate boundary between the nitrogen-affected zone and the unaffected core.

The nitrogen distribution map obtained by EDX (Figure 7B) confirms that nitrogen is concentrated primarily in the near-surface region and decreases progressively with depth, consistent with diffusion-controlled nitriding kinetics. This qualitative distribution is quantitatively supported by the nitrogen depth profile in Figure 7C, where the nitrogen content reaches approximately 5 mass% at the surface and decreases steeply within the first few micrometers, falling below 1 mass% at depths greater than ∼10 μm and approaching zero at around 15 μm. The slight fluctuation observed at intermediate depths can be attributed to local microstructural heterogeneity and phase-dependent nitrogen solubility.

Figure 7D presents the corresponding phase map across the cross-section, providing direct evidence of nitrogen-induced phase transformation. In the near-surface region with high nitrogen content, the microstructure consists of a fine, continuous two-phase mixture of γ (austenite) and α′ (martensite). The formation of the γ phase is attributed to nitrogen dissolution in the α′ matrix, which induces lattice expansion and stabilizes austenite. With increasing depth and decreasing nitrogen content, the stability of the γ phase diminishes. Consequently, beyond approximately 10 μm, the γ phase appears only as isolated or coarse regions embedded within an α′ matrix, rather than as a continuous network.

Overall, Figure 7 demonstrates that the nitrided layer exhibits a pronounced structural and phase gradient governed by nitrogen diffusion. The gradual decrease in nitrogen concentration with depth directly controls the extent of martensite-to-austenite transformation, resulting in a layered microstructure that transitions from a nitrogen-rich, refined γ + α′ structure at the surface to a predominantly α′ structure in the substrate.

### Research on the nitriding behavior of different types of steel

The chemical composition of steel, particularly the type, content, and distribution of strong nitride-forming elements, fundamentally governs its nitriding behavior, phase evolution, and resultant mechanical and functional properties. During GN, variations in alloying systems lead to pronounced differences in nitrogen absorption kinetics, nitride precipitation sequences, phase constitution of the compound layer, diffusion-layer strengthening mechanisms, and the optimal processing window.^108^ A systematic understanding of these compositional effects is therefore essential for accurate process design and performance optimization. This section comparatively analyzes the nitriding behavior of several representative steels subjected to the TGN process, with emphasis on phase formation and structure-property relationships.

#### Nitriding characteristics of bearing steel (GCr15, AISI 52100)

In bearing steels such as GCr15, chromium (Cr) plays a dominant role in controlling nitriding behavior. At appropriate nitriding temperatures, nitrogen diffuses into the surface and reacts preferentially with Cr to form finely dispersed CrN or Cr_2_N precipitates within the ferritic diffusion zone.^129^ These nanoscale alloy nitrides effectively impede dislocation motion, resulting in pronounced precipitation strengthening and improved load-bearing capacity.^130^

From a phase evolution perspective, nitrided GCr15 typically exhibits a well-defined gradient structure. The surface region develops a compound layer consisting primarily of ε-Fe_2-3_N and γ′-Fe_4_N phases, which provides high surface hardness and wear resistance. Beneath this layer, the diffusion zone contains a high density of Cr-rich nitrides embedded in a nitrogen-supersaturated ferrite matrix, contributing to improved subsurface strength and rolling contact fatigue resistance.^131^ The thickness and phase balance of these layers are highly sensitive to nitriding temperature, nitrogen potential, and treatment duration.

#### Nitriding behavior of tool steels (H13, D2)

Tool steels such as H13 and D2 possess complex alloy systems containing multiple strong nitride-forming elements, including Cr, Mo, V, and W. These elements exhibit distinct nitrogen affinities and precipitation kinetics, leading to a multi-stage nitride formation process during GN. Among them, V and Mo display particularly strong tendencies to form stable nitrides at relatively low nitrogen activities.^132^

During nitriding, VN, Mo_2_N, and Cr_2_N precipitate sequentially or concurrently within the diffusion layer, depending on local nitrogen concentration and temperature. VN contributes exceptionally high hardness and thermal stability, Mo_2_N enhances secondary hardening while mitigating brittleness, and Cr_2_N provides baseline strengthening and structural support.^133^ The synergistic precipitation of these nitrides produces a diffusion layer with superior hardness retention at elevated temperatures, making nitrided tool steels particularly suitable for hot-working and high-load applications. Excessive nitride precipitation, however, may lead to embrittlement, highlighting the need for precise process control.

#### Nitriding characteristics of stainless steels (420, 304)

GN of stainless steels presents distinct challenges due to the presence of a stable chromium-rich passive film (mainly Cr_2_O_3_), which acts as an effective diffusion barrier and significantly suppresses nitrogen adsorption, dissociation, and inward diffusion.^79^ Consequently, appropriate surface activation pretreatments are indispensable prerequisites for achieving effective nitriding. Common pretreatment strategies include chemical or electrochemical pickling to remove surface oxides, mechanical activation (e.g., polishing or shot peening) to disrupt the passive film, and plasma-assisted activation, in which energetic ions sputter the oxide layer and generate a highly reactive surface. These pretreatments not only eliminate or thin the passive film but also increase surface defect density and adsorption sites, thereby enhancing nitrogen uptake and facilitating subsequent diffusion into the substrate.

From a phase-formation standpoint, the high chromium content in stainless steels promotes the precipitation of CrN and Cr_2_N during nitriding, which can markedly improve surface hardness and wear resistance. However, excessive chromium nitride precipitation may lead to local Cr depletion in the matrix, undermining the stability of the passive film and thus deteriorating corrosion resistance.^134^ In martensitic stainless steel 420, nitriding typically results in the formation of alloy nitrides within the diffusion zone, providing significant strengthening but requiring strict control of nitriding temperature and nitrogen potential to limit detrimental Cr depletion. Under low-temperature nitriding conditions, nitrogen can also dissolve interstitially into the martensitic lattice, leading to the formation of expanded martensite (α′N), a nitrogen-supersaturated metastable phase analogous to expanded austenite. This phase is characterized by lattice expansion, high compressive residual stresses, and enhanced surface hardness, while suppressing extensive chromium nitride precipitation and thereby mitigating corrosion degradation. In contrast, in austenitic stainless steel 304, low-temperature nitriding (generally below ∼450 °C) favors the formation of expanded austenite (γN or S-phase), a metastable nitrogen-supersaturated phase characterized by lattice expansion, high compressive residual stresses, and exceptional hardness, while largely preserving chromium in solid solution and maintaining corrosion resistance.^135^

#### Nitriding advantages of special case-hardening steels (41CrAlMo7)

Specially developed nitriding steels, such as 41CrAlMo7, are designed to maximize nitriding efficiency through tailored alloy compositions. A defining feature of these steels is the intentional addition of aluminum (Al), a strong nitride-forming element with a high affinity for nitrogen. During GN, Al reacts with nitrogen to form finely dispersed AlN precipitates with nanoscale dimensions and extremely high number density.^136^

These AlN precipitates provide exceptionally strong precipitation strengthening and exhibit outstanding thermal stability, resisting coarsening even at elevated service temperatures. As a result, the nitrided layer maintains its hardness and load-bearing capability over prolonged service periods.^137^ The combination of a stable compound layer and a robust diffusion zone enables special nitriding steels to perform reliably under complex stress states, making them ideal for critical components operating under demanding conditions.

Overall, the nitriding behavior of steels is fundamentally dictated by alloy composition, particularly the nature and concentration of strong nitride-forming elements. Differences in phase formation, nitride precipitation behavior, and diffusion-layer evolution directly determine the mechanical and functional performance of the nitrided layer.^138^ In practical applications, nitriding parameters must be carefully tailored to the specific steel grade to fully exploit the potential of GN and achieve optimal surface performance.

### Research on corrosion resistance and wear resistance mechanisms of the nitrided layer

The wear resistance of the nitrided layer is one of its most critical engineering characteristics. Its excellent wear resistance arises from the synergistic action of multiple mechanisms. A thorough understanding of these mechanisms is essential for optimizing process parameters and expanding application fields.

### Research on the wear resistance mechanisms of the nitrided layer

**Figure 8:**
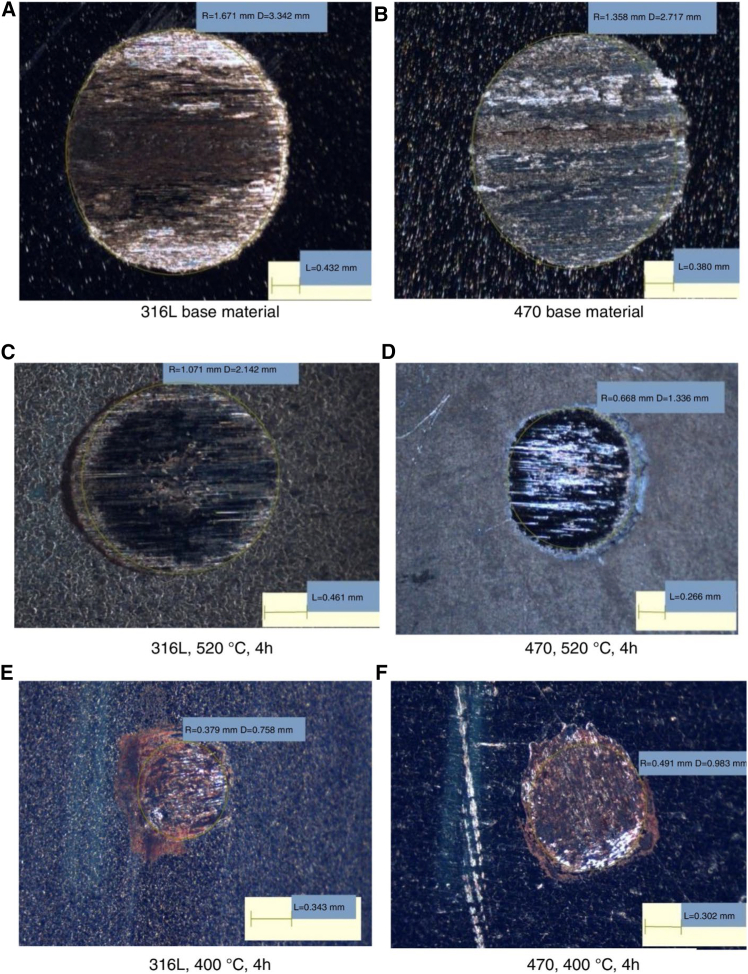
Optical microscope image after micro-abrasive wear test on AISI 316L and AISI 470 steel with different nitriding treatment Crater of (A) AISI 316L base material. (B) AISI 470 base material. (C) AISI 316L treated at 520°C for 4h. (D) AISI 470 super-ferritic steel treated at 520°C for 4h. (E) AISI 316L treated at 400°C for 4h. (F) AISI 470 super-ferritic treated at 400°C treated for 4h (reprinted from Araújo et al.^139^ Copyright 2019, with permission from Elsevier.

Nitriding, as an efficient surface strengthening technology, primarily enhances wear resistance by simultaneously resisting abrasive and adhesive wear. Figure 8 presents optical microscopy images of caps after micro-abrasive wear tests on AISI 316L and AISI 470 steels, with and without thermochemical treatment. As shown in all images, the wear mechanism is characterized by a combination of scratching and rolling. The wear of samples treated by GN is lower than that of untreated samples.^139^

For abrasive wear, the superiority of the nitrided layer arises not only from increased surface hardness but, more importantly, from its unique microstructural strengthening mechanisms.^140^ When the surface layer forms a dispersion of alloy nitrides, these nanoscale hard phases effectively hinder particle embedment and plowing. In V-containing alloy steels, VN maintains a coherent relationship with the matrix, enhancing strain-hardening capacity while increasing hardness. As a result, the nitrided layer exhibits higher elastic recovery during wear, significantly slowing the accumulation of plastic deformation.^141^

For adhesive wear resistance, the ε-Fe_2-3_N phase on the nitrided surface plays a critical role. This phase has an HCP crystal structure, markedly different from that of common metallic matrices. This crystallographic incompatibility effectively suppresses atomic diffusion and adhesive tendencies between friction pairs.^142^ Further research shows that during continued friction, the ε phase develops crystallographic orientations with slip planes parallel to the friction surface, thereby reducing the friction coefficient. Meanwhile, friction-induced thermal effects may lead to the formation of an oxygen-rich surface layer with self-lubricating properties, further improving friction conditions.^143^

### Research on the corrosion resistance mechanism of the nitrided layer

Nitriding treatment significantly enhances not only the material’s wear resistance but also its corrosion resistance by altering the surface microstructure and chemical composition.^144^ The compound layer, acting as the outermost layer after nitriding, provides primary corrosion protection by forming a passivation film in corrosive environments. Studies have shown that the passivation film formed on the surface of a compound layer dominated by the ε phase exhibits a distinct bilayer structure: the outer layer is primarily composed of Fe_3_O_4_, whereas the inner layer contains nitrogen-rich Fe_3_O_4_ and Fe_2_O_3_ oxides. This structure markedly increases the density and stability of the passivation film and confers excellent self-healing capability, thereby providing continuous and effective corrosion protection.^145^^,^^146^

**Figure 9:**
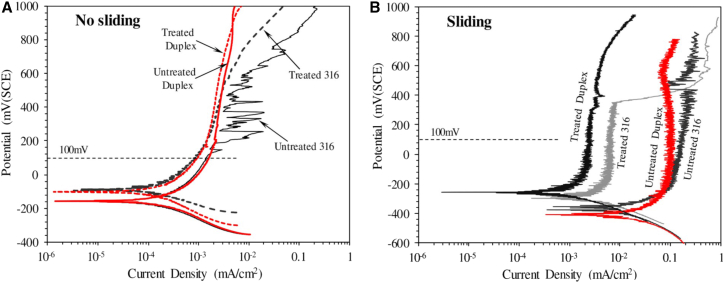
Dynamic point polarization curves of different specimens in 3.5% NaCl solution (A) Without sliding and (B) with sliding (2 N, 2 Hz) (reprinted from Haruman et al.^147^ Copyright 2020, with permission from Elsevier).

From an electrochemical perspective, the nitrided layer substantially improves the electrochemical behavior of materials in corrosive environments. Figure 9 compares the tribocorrosion behavior of gas-nitrided austenitic (316L) and duplex (2205) stainless steels treated at 450 °C for 10 h. In typical corrosive environments, the corrosion potential of the nitrided layer shifts markedly in the positive direction compared with the substrate, and the corrosion current density is substantially reduced.^147^ This improvement is primarily attributed to the high nitrogen content in the ε phase, which modifies the semiconductor properties of the surface oxide, changing it from p-type to n-type. This transition increases the pitting breakdown potential and enhances resistance to localized corrosion.^148^

The improvement of corrosion resistance by nitriding also manifests in eliminating microscopic electrochemical heterogeneity. Conventional steels readily form micro-corrosion cells due to the large electrode potential differences between carbide and ferrite phases.^149^ High-quality nitriding produces compound layers with highly uniform chemical composition and crystal structure, fundamentally eliminating microscopic electrochemical heterogeneities.^150^^,^^151^

It is noteworthy that the influence of nitriding on the corrosion resistance of different steel grades varies substantially. For example, under low-temperature nitriding with strictly controlled temperature, the S-phase expanded layer formed in austenitic stainless steel can simultaneously enhance surface hardness and corrosion resistance.^152^^,^^153^ However, improper temperature control can induce chromium nitride precipitation, resulting in chromium depletion in the matrix and thus deteriorating the inherent corrosion resistance. This phenomenon underscores the necessity of precise control over process parameters during nitriding, as only targeted optimization enables the synergistic enhancement of surface hardness and corrosion resistance.^154^^,^^155^

### Comparison and application optimization of corrosion and wear resistance mechanisms

As discussed earlier, nitriding achieves a synergistic enhancement of wear and corrosion resistance through distinct underlying mechanisms. However, these mechanisms differ markedly in their modes of action, performance characteristics, and applicable service conditions. To provide a clear understanding of their core features, Table 4 systematically compares the mechanisms, key advantages, limitations, and typical application fields of different wear- and corrosion-resistance mechanisms. The comparison highlights three wear-resistance mechanisms—abrasive wear, adhesive wear, and fatigue wear—and two corrosion-resistance mechanisms: passivation film formation and composition homogenization. This provides guidance for optimizing technical solutions according to specific service conditions.

**Table 4:** Comparative analysis of different corrosion and wear resistance mechanisms

Wear/Corrosion mechanism	Action mechanism	Advantages	Disadvantages	Application field
Abrasive wear resistance	Relies on dispersed ultra-high hardness nitrides to resist the indentation and plowing of hard particles.	Significant increase in surface hardness, extremely strong resistance to abrasive wear.	Excessive nitrides or overly thick compound layers increase brittleness, prone to microspalling under heavy impact.	Mining machinery parts, injection molding machine screws, extrusion dies, and so forth, with severe abrasive wear conditions.
Adhesive wear resistance	Surface ε phase has an HCP structure with few slip systems, not prone to atomic-level welding with counter bodies.	Fundamentally reduces friction coefficient, prevents “galling” or “scuffing.”	Compound layer brittleness becomes apparent when too thick, prone to failure under impact or point contact.	Components with relative sliding friction pairs, such as crankshafts, camshafts, and cylinder liners.
Passivation film formation	Compound layer forms a dense oxide film acting as a physical barrier to isolate corrosive media.	Quick, economical protection, effective in uniform corrosion environments.	Poor self-repair ability of passivation film, susceptible to pitting in Cl^−^ containing environments.	General mechanical parts, hydraulic rods, and structural parts working in mildly corrosive environments.
Composition homogenization	Eliminates microscopic electrochemical heterogeneity, preventing the formation of local corrosion cells.	Provides uniform protection, effectively resists pitting and grain boundary corrosion.	Protection strictly depends on the density and continuity of the compound layer.	Fuel system components, injection molding machine screws, and applications requiring resistance to localized corrosion.

Although the individual mechanisms of corrosion and wear are relatively well understood, a systematic and quantitative understanding of their synergistic effects under real service conditions remains insufficient. Under extreme conditions involving high temperature, corrosive media, mechanical impact, and other coupled fields, the material failure behavior and dominant mechanisms may fundamentally change, and existing theoretical models are still difficult to accurately predict this. In addition, the mechanisms of corrosion initiation and crack propagation at microscopic defects such as phase and grain boundaries, as well as the deformation and failure mechanisms of nanoscale nitrides under complex stress states, involve numerous fundamental scientific questions that remain unresolved. Therefore, further in-depth research is required to enable controllable processing and reliable engineering application of nitrided materials under extreme conditions.

## Catalytic gas nitriding

Although TGN technology significantly enhances the corrosion and wear resistance of steel, the process typically requires a long processing time, which severely limits production efficiency. To overcome this limitation, researchers have combined advanced surface engineering technologies with TGN, thereby enhancing nitriding efficiency and improving the microstructural characteristics of the nitrided layer. Subsequent chapters will introduce these advanced technologies for auxiliary GN.

### Surface pretreatment techniques

Surface pretreatment plays a crucial role in the GN process. Optimizing the surface condition of workpieces creates favorable conditions for subsequent nitriding, significantly enhancing efficiency, improving layer quality, and ensuring uniformity and reproducibility. Pretreatment produces highly active, high-energy metal surfaces that promote the adsorption and dissociation of nitrogen atoms. In addition, pretreatment induces mechanical activation and structural optimization, creating pathways and driving forces for the rapid diffusion of nitrogen atoms. Surface pretreatment removes oxide films and contaminants from the workpiece and introduces lattice defects, resulting in an unstable metal surface with high free energy. The high surface activity markedly reduces the activation energy required for the chemisorption of ammonia molecules and their stepwise dissociation into atomic nitrogen, thereby greatly enhancing the generation efficiency of surface nitrogen atoms, similar to a catalytic effect. The rapid increase in surface nitrogen concentration establishes a steep concentration gradient between the surface and the interior of the workpiece, providing the primary driving force for the continuous and rapid diffusion of nitrogen atoms into the substrate.^156^^,^^157^^,^^158^ Ultimately, this process yields a nitrided layer with sufficient depth, appropriate hardness, and uniform microstructure. This reduces component deformation, ensures consistent surface conditions across the entire part, and guarantees overall performance.

### Surface nanocrystallized gas nitriding

**Figure 10:**
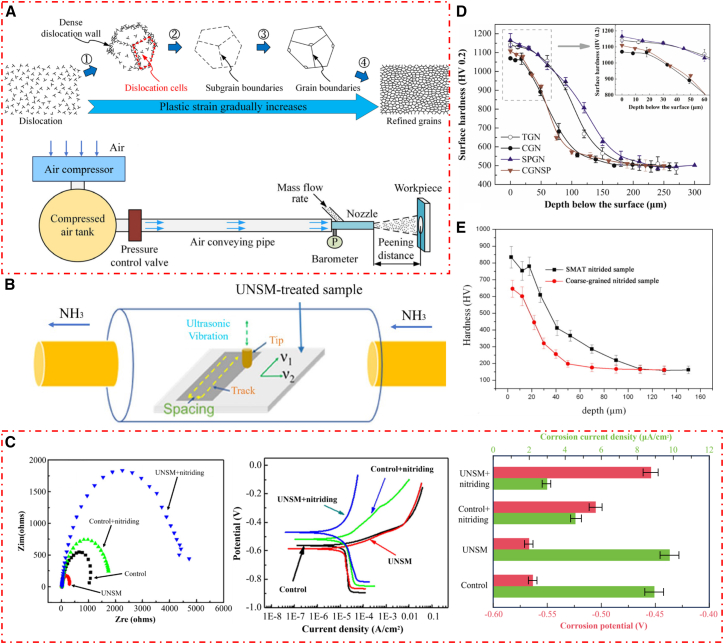
Effects of surface nanocrystallization on steel properties (A) Schematic of grain refinement mechanism after SPD and working principle of shot peening device (reprinted from Lin et al.^159^ Copyright 2022, with permission from Elsevier). (B) Mechanism diagram of UNSM. (C) Nyquist curves, polarization curves, corrosion current density, and corrosion potential of nitrided samples in a 3.5 wt % NaCl solution under different experimental conditions (reprinted from Zhao et al.^165^ Copyright 2019, with permission from Elsevier). (D) Microhardness profiles of nitrided samples under varying processing conditions, test load:200g, dwelling time:10 s (reprinted from Liu et al.^166^ Copyright 2019, with permission from Elsevier). (E) Microhardness distributions of nitrided samples treated by different surface modification methods, test load:15g, dwelling time:10 s (reprinted from Sun et al.^167^ Copyright 2016, with permission from Elsevier).

Surface nanocrystallization is an advanced surface modification technique, primarily achieved by inducing severe plastic deformation (SPD) in the metal surface layer through specialized mechanical treatments.^159^ Its physical essence is to introduce crystal defects such as dislocations, twins, and sub-grain boundaries into the near-surface region of the material by utilizing external mechanical energy.^160^ These defects interact, leading to continuous subdivision and refinement of the original coarse grains. Ultimately, these processes produce a nanostructured layer, as illustrated by the grain refinement process in Figure 10A.^161^ A key feature of this method is the transformation of the surface layer from a coarse-grained to a nanocrystalline structure, without changing the chemical composition or phase constitution of the bulk material.^162^ This technique has been successfully applied to various metallic materials, such as pure iron, low-carbon steel, stainless steel, pure zinc, titanium, and titanium alloys.^163^ Common surface nanocrystallization methods include ultrasonic nanocrystal surface modification (UNSM), shot peening (SP), and surface mechanical attrition treatment (SMAT).^164^

As an emerging technique, UNSM employs ultrasonic-frequency impacts on the specimen surface, inducing substantial SPD (mechanism shown in Figure 10B).^165^ Studies have demonstrated that UNSM refines surface grains to the nanoscale, producing nanocrystalline layers up to 200 μm in depth under optimized parameters.^168^ The advantages of UNSM include not only generating SPD but also significantly improving surface finish by combining ultrasonic shot peening with polishing.^169^^,^^170^ For example, Ma et al.^171^ reported that UNSM treatment markedly reduced the surface porosity of additively manufactured NiTi alloy. Recent studies have further confirmed that UNSM substantially enhances nitriding efficiency. Zhao et al.^165^ found that when UNSM was used as a pretreatment process for GN (450°C, 6h), the composite-treated samples showed better corrosion resistance (Figure 10C). The severe plastic deformation induced by UNSM generated a high density of crystal defects in the surface layer, providing rapid diffusion pathways for nitrogen atoms during subsequent nitriding. This facilitated the formation of a thicker and denser nitride layer enriched in ε-Fe_2-3_N and γ′-Fe_4_N. This high-quality protective barrier effectively inhibits the penetration of corrosive media and delays pitting initiation, enabling the UNSM+GN composite process to achieve the highest corrosion potential, the lowest corrosion current density, and the most favorable electrochemical impedance response.^172^

SP is a surface modification technique that induces plastic deformation in the surface layer of a material by using a high-velocity stream of peening media. This process generates a gradient residual compressive stress field and microstructural refinement in the near-surface region, thereby markedly improving the fatigue limit and wear resistance of components.^173^ Liu et al.^166^ investigated the effects of the combined SP + GN surface treatment on the surface properties of H13 steel. The hardness profiles of samples subjected to different processing conditions are shown in Figure 10D. Samples pretreated with SP exhibited the highest hardness, likely due to compound layer formation and microdefects that accelerate nitrogen diffusion and promote nitrided layer growth. Similarly, Lin et al.^19^ reported the influence of the SP + GN composite process on subsurface residual compressive stress (RCS) in steel. They found that the residual compressive stress field effectively suppressed fatigue crack initiation and propagation. This stabilizing effect allows the steel to retain a more stable compressive stress state during high-temperature service.

SMAT similarly employs SPD to produce a nanostructured surface layer on steel.^174^^,^^175^ Studies have shown that SMAT-treated samples possess nanostructures with numerous grain boundaries, which markedly enhance nitrogen diffusion compared with coarse-grained materials.^176^^,^^177^ Sun et al.^167^ investigated the effects of the combined SMAT+GN at 520 °C for 6 h on the microstructure and surface hardness of pure iron, as illustrated in Figure 10E. The combined SMAT+GN samples exhibited a distinctive nitride layer morphology. Compared with the AR samples, the composite treatment samples developed a thicker compound layer and a more pronounced diffusion zone. This strengthening approach offers a new pathway for developing iron-based materials with enhanced surface performance and overall mechanical properties.

### Laser-assisted gas nitriding

**Figure 11:**
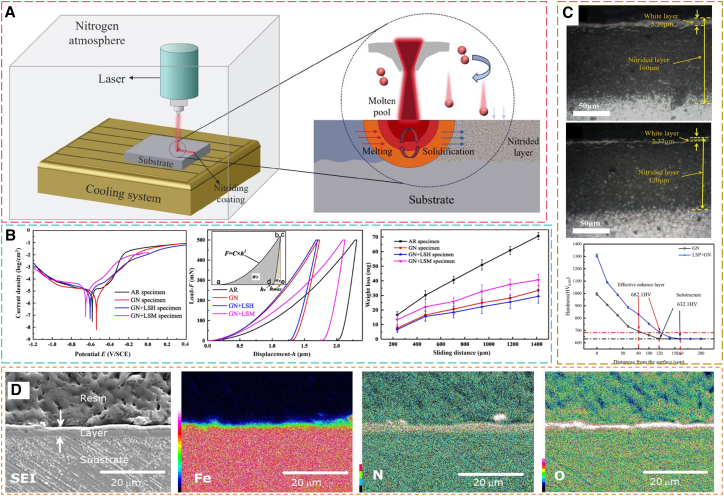
Effects of LGN on steel performance (A) Schematic of LGN (reprinted from Liu et al.^178^ Copyright 2024, with permission from Elsevier). (B) Potentiodynamic polarization curves of the specimens in 3.5% NaCl solution, load-displacement curves, and weight loss vs. sliding distance for nitrided samples under different treatment conditions, test load: 500 mN, holding time: 5s (reprinted from Yan et al.^17^ Copyright 2020, with permission from Elsevier). (C) Cross-sectional micrographs of LSP + GN and GN samples, test load: 100g, loading time: 15s (reprinted from Luo et al.^185^ Copyright 2023, with permission from Elsevier). (D) SEM image and EDS analysis of the surface of AISI 316 stainless steel treated by LSM + GN (reprinted from Ohtsu et al.^186^ Copyright 2024, with permission from Elsevier).

Laser-assisted gas nitriding (LGN) is an efficient surface alloying technology based on the synergistic action of high-power density laser beams and nitrogen-containing atmospheres.^178^ This method subjects workpiece surfaces to ultra-rapid thermal cycles, lasting from milliseconds to seconds, through high-energy laser irradiation.^179^ In a protective atmosphere, nonlinear interactions between laser-induced plasma and the material surface enable energy coupling, promoting thermal diffusion and [N] chemisorption. Careful control of processing parameters is essential: laser power density determines the temperature field in the molten pool; scanning speed controls the thermal interaction time; and spot diameter with overlap ratio together regulate overlapping effects in the heat-affected zone.^180^^,^^181^^,^^182^ Coordinated adjustment of these parameters allows precise control of nitriding case depth, from micrometers to millimeters, meeting gradient hardening requirements for various service conditions. The primary advantage is the laser’s ultrahigh heating and cooling rate, which creates a non-equilibrium thermal process, suppressing nitride coarsening and promoting metastable microstructure formation in the surface layer.^22^ Compared with TGN, the rapid solidification in LGN (Figure 11A) effectively inhibits nitride precipitation at grain boundaries. Additionally, the combined effects of surface densification and RCS impart ultrahigh hardness and outstanding wear resistance to the modified layer.^183^^,^^184^

Existing studies have confirmed that laser irradiation within a flowing nitrogen atmosphere constitutes a viable strategy for enhancing surface quality in nitrided samples. This process relies on both the laser-induced thermal effects and the nitriding reactions occurring at the substrate and nitrogen gas interface.^187^^,^^188^ Consequently, a significant number of researchers have attempted to combine laser surface treatment with GN to achieve a superior surface layer. Yan et al.^17^ investigated the effect of GN (550°C, 20h, K_N_ = 0.18) combined with laser surface melting (LSM) and laser surface hardening (LSH) on the properties of P20 steel, as shown in Figure 11B. The GN + LSH composite surface treatment significantly enhances the wear resistance of the nitrided layer, primarily attributed to the solid solution strengthening and Hall-Petch strengthening induced by the martensite formed during the LSH process. However, the difference in electrochemical properties between the retained austenite and martensite in the microstructure can induce interfacial galvanic corrosion, which in turn leads to a decrease in corrosion resistance. In contrast, the GN + LSM composite treatment is attributed to denitriding of the nitrided layer induced by high-temperature melting, resulting in the formation of void defects at the bottom of the solidification layer. This consequently leads to a surface layer with unexpectedly lower hardness and wear resistance compared to the solely nitrided layer. Conversely, Luo et al.^185^ investigated the influence of LSP on the surface properties of nitrided H13 steel, revealing that LSP post-treatment significantly refines near-surface grains and enhances wear resistance by promoting nitrogen diffusion along grain boundaries. As shown in Figure 11C, the LSP+GN composite specimen exhibits a significantly thickened white layer and diffusion zone, accompanied by increased microhardness and effective hardened layer thickness due to the synergistic effect of grain refinement and enhanced nitrogen permeation. Additionally, Ohtsu et al.^186^ reported that a nitrogen-saturated layer with a thickness of 3 μm was formed on AISI 316 stainless steel under 4 W laser irradiation. This layer prevents plastic deformation and wear, thereby improving the wear resistance of AISI 316 stainless steel. The SEM image (Figure 11D) confirmed the presence of a multi-layered structure on the surface, comprising a thin, submicron-thick uppermost layer and a 3 μm underlying layer. The uppermost layer contained iron and oxygen atoms, whereas nitrogen was nearly undetectable. In contrast, nitrogen was predominant in the underlying layer, with oxygen being virtually absent. These results demonstrate that the combination of laser irradiation with gaseous nitrogen enables the formation of a nitrogen-saturated layer on the steel surface, thus enhancing its surface properties, such as wear resistance.

### Pre-oxidized gas nitriding

**Figure 12:**
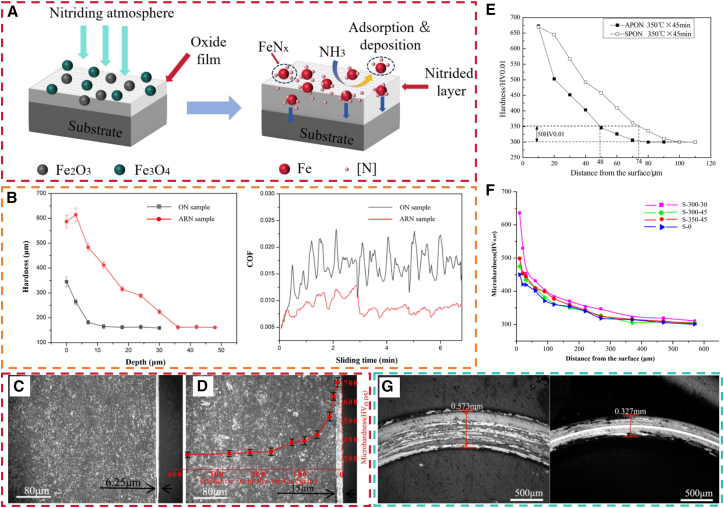
Pre-oxidized gas nitriding of steel (A) Schematic mechanism of POGN. (B) Surface hardness and COF of nitrided samples with vs. without PO treatment, test load:50N, sliding speed: 5 mm/s, sliding amplitude (L): 10 mm (reprinted from Hu et al.^196^ Copyright 2023, with permission from Elsevier). (C) Cross-sectional microstructure of GN samples. (D) Cross-sectional microstructure and microhardness profile of POGN samples pre-oxidized at 300°C for 30min, test load:50g, holding duration:15s (reprinted from Li et al.^197^ Copyright 2014, with permission from Elsevier). (E) Microhardness of samples nitrided at 560 °C for 2h using two pre-oxidation techniques, test load:10 g, holding duration:15s (reprinted from Peng et al.^198^ Copyright 2019, with permission from Elsevier). (F) Surface microhardness of nitrided samples under different PO parameters, test load:50 g, holding duration:15s (reprinted from Liu et al.^189^ Copyright 2014, with permission from Elsevier). (G) Microscopic wear scar morphology of AR and POGN samples, test load:200 g, milling time:10 min (reprinted from Tang et al.^199^ Copyright 2020, with permission from Elsevier).

Pre-oxidized gas nitriding (POGN) consists of a standardized pre-oxidation step prior to GN, during which a dense oxide film forms on the workpiece surface. This film facilitates the permeation of [N].^189^ This process shortens the nitriding cycle by 15–30% while maintaining the desired nitriding quality, thereby significantly improving productivity and reducing energy consumption.^190^^,^^191^ Two main mechanisms have been proposed to explain POGN. The first theory suggests that a compact Fe_3_O_4_ oxide film forms on the surface and is subsequently reduced by hydrogen during the initial nitriding stage, as shown in Equation 14.^192^ This reduction generates a clean surface with numerous active sites for nitrogen adsorption, thereby enhancing the substrate’s ability to absorb nitrogen.^193^ In contrast, the second theory argues that the inherently porous pre-oxidized surface increases the specific surface area available for the reaction between NH_3_ and the substrate, thereby enlarging the effective reaction zone.^194^ Concurrently, the reaction between NH_3_ and Fe_3_O_4_ (Equation 15) promotes the formation of additional pores. This reaction enhances ammonia adsorption and facilitates its decomposition on the surface, releasing N atoms. The reaction mechanism is illustrated in Figure 12A. Furthermore, the oxygen generated from this reaction can be adsorbed and subsequently diffuse into the substrate, promoting oxynitriding. Importantly, oxygen infiltration exerts a catalytic effect on the nitriding reaction.^195^

(14) $${\text{Fe}}_{3}O_{4}+4H_{2}=3\text{Fe}+4H_{2}O$$

(15) $$4{\text{Fe}}_{3}O_{4}+4{\text{NH}}_{3}=4{\text{Fe}}_{3}N+6H_{2}O+5O_{2}$$

The POGN process is cost-effective and markedly improves the homogeneity of the nitrided layer. Consequently, this technique has been widely combined with conventional nitriding methods by many researchers.^199^ Wen et al.^196^ developed an anodic oxidation-reduction composite treatment that effectively induced nanocrystallization on the surface of pure iron. Subsequent experiments revealed the formation of a composite layer approximately 3 μm thick, composed of Fe_3_N and Fe_4_N phases, on the surface of POGN samples. Compared with non-pre-oxidized nitrided samples, POGN samples exhibited higher surface hardness and a lower COF (Figure 12B). Li et al.^197^ investigated the effect of POGN on the surface properties of AISI 4140 steel. Samples were nitrided at 500°C for 4h. The cross-sectional microstructures and microhardness profiles of AISI4140 steel with and without pre-oxidation are shown in Figure 12C and d, respectively. The maximum compound layer thickness of 15 μm was obtained after nitriding at 500°C for 4h under the PO condition of 300°C and 30min. The results demonstrate that the nitrided layer thickness in POGN samples was doubled. Peng et al.^198^ further investigated the effects of salt-bath pre-oxidation (SPO) and air pre-oxidation (APO) on the nitriding behavior of AISI 1045 steel. Both methods enhanced the microhardness of the steel, but SPO yielded superior enhancement compared to APO (Figure 12E). This finding provides a theoretical basis for exploring new pre-oxidation techniques and advancing the development of POGN. Building on this work, Liu et al.^189^ examined the influence of pre-oxidation parameters on the GN behavior of AISI4140 steel. As shown in Figure 12F, samples pre-oxidized at 300°C for 30min exhibited the highest microhardness, with a nitrided layer thickness more than twice that of the AR samples. Collectively, these results demonstrate that under identical processing conditions, pre-oxidation treatment enhances both the probability and capability of [N] adsorption on the sample surface. Furthermore, it establishes a steeper nitrogen concentration gradient during the initial nitriding stage, thereby increasing the diffusion activation energy for [N] and ultimately accelerating nitriding kinetics.^200^^,^^201^

### Surface-active catalyzed gas nitriding

In addition to the aforementioned surface pretreatment techniques, active catalyzed treatment applied to the steel surface is also a commonly used method to enhance the efficiency of GN. Surface-active catalyzed GN alters the decomposition kinetics of NH_3_, optimizes surface reaction pathways, and creates highly active diffusion channels, thereby significantly improving both the efficiency of nitriding and the quality of the nitrided layer. For instance, using rare-earth elements as catalysts can effectively reduce the activation energy for NH_3_ decomposition, thereby promoting the generation of [N]. A nanoscale metal coating (such as Ni, Ti, Cr, and so forth) is pre-deposited on the surface of the steel substrate, and GN is catalyzed through physical and chemical reactions to construct a highly active surface layer. Furthermore, introducing carbon-containing gases (CO_2_, CH_3_OH) into the nitriding atmosphere can significantly enhance the efficacy of GN by leveraging C-N synergistic interactions. These catalyzed techniques will be comprehensively discussed in subsequent sections.

### Rare-earth catalyzed gas nitriding

**Figure 13:**
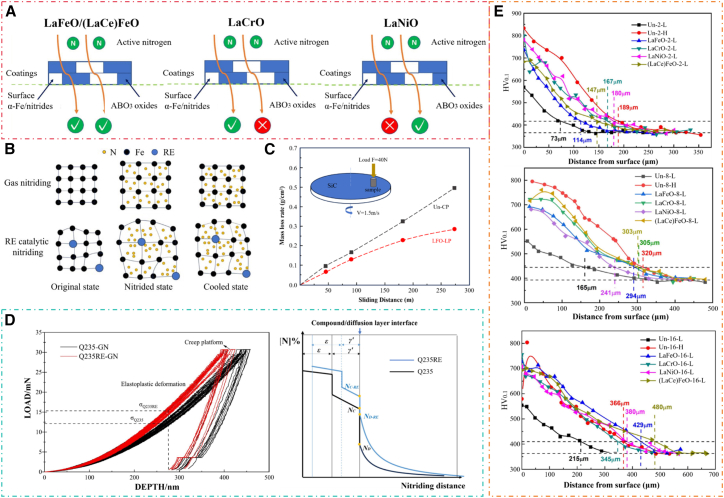
Schematic of RE catalytic mechanisms and effects on nitrided layers (A) Internal diffusion of [N] (reprinted from Zhang et al.^150^ Copyright 2021, with permission from Elsevier). (B) Crystal lattice diagrams of diffusion layer in Q235 and Q235RE during nitriding (reprinted from Zhao et al.^206^ Copyright 2024, with permission from Elsevier). (C) Wear resistance comparison between RE-catalyzed and un-catalyzed samples (reprinted from Li et al.^207^ Copyright 2023, with permission from Elsevier). (D) Displacement-load curves of GN and RE + GN samples with corresponding [N] concentration profiles in nitrided layers, test load: 300g, holding time: 15s.^206^ (E) Microhardness distribution of nitrided samples under varying nitriding durations, test load: 100g, holding time: 15s.^150^

Rare-earth (RE) catalyzed gas nitriding improves performance by using RE elements as highly efficient catalysts during nitrogen permeation. The RE catalysts most commonly applied are ABO_3_-type perovskite oxides, including LaFeO_3_, LaCrO_3_, and LaNiO_3_.^23^ RE elements are widely recognized to promote high-concentration nitride formation or sustain high K_N_ conditions, thereby enhancing N atom adsorption and diffusion into the substrate.^202^^,^^203^ First-principles calculations by Yang et al.^204^ showed that RE doping markedly lowers the diffusion energy barrier of N atoms on steel surfaces. You et al.^205^ proposed the “RE catalyzing anti-trap” theory, which explains that strong repulsive forces between RE and N atoms act as the main driving force for lowering diffusion barriers. Moreover, Zhang et al.^150^ demonstrated that the internal diffusion of N atoms is another critical factor influencing the catalytic activity of ABO_3_-type oxides. As shown in Figure 13A, [N] migrates through multiple diffusion pathways across ABO_3_/α-Fe interfaces in both LaFeO_3_ and (La,Ce)FeO_3_ coatings. By contrast, N diffusion in LaCrO_3_ and LaNiO_3_ coatings is restricted to a single pathway, occurring solely through either the ABO_3_ oxide or the adjacent α-Fe phase. This restriction of diffusion pathways markedly slows the nitriding kinetics of LaCrO_3_- and LaNiO_3_-coated samples. Figure 13B illustrates the lattice evolution within the diffusion layer at different nitriding stages for both Q235 and Q235RE steels.^206^ Compared with AR samples, RE samples show more pronounced volumetric contraction during cooling, leading to higher surface RCS in their compound layer. Notably, the RE sample compound layer exhibits both elevated RCS and marked grain refinement. The synergy of these factors substantially improves the plasticity and toughness of the RE sample compound layer.

However, the existing forms of RE species have not been stably detected within the formed nitrided layers to date. The discrepancy between theoretical predictions and experimental observations of RE-catalyzed GN highlights the need for further investigation of its core mechanisms. Nevertheless, this process undeniably enhances the corrosion and wear resistance of nitrided samples. Li et al.^207^ investigated the effects of LaFeO_3_ on LPGN of M50NiL steel, with wear test results shown in Figure 13C. Uncatalyzed samples nitrided at conventional pressure (Un-CP) exhibited substantial wear, whereas the mass loss of low-pressure catalyzed samples (LFO-LP) was reduced to 50% of that of Un-CP. This marked difference results from the catalytic effect of LaFeO_3_, which promotes the decomposition of NH_3_, perturbs the surface nitrogen equilibrium, and thereby accelerates nitriding kinetics. Meanwhile, Zhao et al.^206^ also investigated the effect of RE catalyzed GN (520°C, 3h) on the surface properties of Q235 steel. Figure 13D illustrates the surface nitrogen concentration of both sample types during nitriding. Consistent with previous studies, the RE-catalyzed samples exhibited higher nitrogen potential, resulting in a steeper K_N_ gradient that accelerated [N] diffusion. Subsequent experiments confirmed the enhanced wear resistance of RE-catalyzed nitrided samples. Zhang et al.^150^ studied the influence of ABO_3_ oxides on the GN of AISI 4140 steel. Figure 13E presents the microhardness of nitrided samples under different conditions. Studies show that ABO_3_ oxides exhibit catalytic activity in LPGN, with LaFeO_3_ and (La_0.5_Ce_0.5_)FeO_3_ displaying pronounced efficacy during prolonged nitriding. Short-term nitriding is mainly driven by K_N_, whereas long-term efficiency is strongly correlated with the active oxygen content on the nitrided surface.

### Alloying elements catalyzed gas nitriding

Recent studies show that pre-depositing specific functional coatings on substrates can effectively catalyze GN, thereby overcoming major efficiency bottlenecks.^208^ These coatings exhibit strong catalytic effects during nitriding due to their distinct physicochemical properties. The catalytic mechanism operates mainly in two ways. First, active metal elements in the coatings generate electron-rich sites, which markedly lower the activation energy for N_2_ dissociation and thus accelerate nitrogen atom generation.^209^^,^^210^ Second, some coatings develop high-density grain boundary networks that both facilitate the rapid diffusion of nitrogen atoms and suppress excessive growth of brittle nitrides.^211^^,^^212^ Experimental studies demonstrate that pre-nickel-plated coatings reduce the nitriding temperature by 50°C–100 °C, shorten processing time by 15–30% under identical conditions, and markedly improve the toughness of the nitrided layer.^213^^,^^214^ A detailed understanding of the catalytic pathways for nitrogen activation and diffusion in these coatings offers new insights for the development of high-efficiency nitriding technologies and has important implications for advancing theoretical innovation in surface reaction engineering.^215^

**Figure 14:**
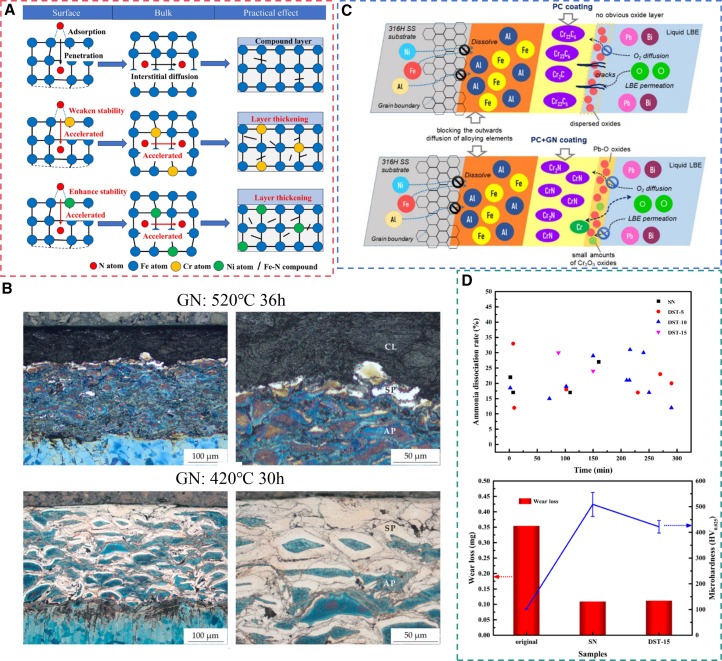
Effects of coating-catalyzed nitriding on steels (A) Schematic of Cr/Ni catalytic nitriding mechanisms (reprinted from Chen et al.^218^ Copyright 2025, with permission from Elsevier). (B) Optical-microscopic images of a Beraha II-etched AISI 316L HVOF coating in as-sprayed conditions after GN with different parameters in overall view and detail with phase declaration (CL: compound layer; SP: S-phase; AP: austenitic phase).^219^ (C) Corrosion mechanisms of PC and PC + GN samples in LBE (reprinted from Guo et al.^25^ Copyright 2024, with permission from Elsevier). (D) Wear test and ammonia dissociation rate changes with nitriding time for each sample, test load: 25g, holding time: 10s.^221^

The core of alloy-element coating-catalyzed nitriding technology lies in understanding the diffusion behavior of N atoms in alloyed materials. It is widely recognized that alloying modifies the electronic structure of the substrate, reduces the activation energy for nitrogen diffusion, and thereby accelerates the nitriding process.^216^^,^^217^ Chen et al.^218^ studied the effects of Cr and Ni doping on nitrogen adsorption, dissolution, and diffusion in α-Fe using combined first-principles calculations and experimental validation. The catalytic mechanism is schematically illustrated in Figure 14A. The results show that both Cr and Ni doping lower the energy barriers for nitrogen migration from the surface to the bulk and for diffusion through octahedral interstitial sites, with Ni having the stronger effect. To verify the catalytic effect of pretreatment on GN, Lindner et al.^219^ studied the influence of HVOF coatings on the GN of AISI 316L. Figure 14B shows the cross-section of the coatings depending on the GN parameters used. Within the coating, three-phase regions were identified, demonstrating a heterogeneous microstructure. Several isolated white particles exhibited greater surface distances, indicating coating permeability. A composite layer thickness exceeding 100 μm also proved that the higher coating porosity favors the GN behavior. Furthermore, Guo et al.^25^ fabricated ∼80 μm chromium carbide/nitride coatings on 316H stainless steel substrates and studied their corrosion behavior in lead-bismuth eutectic (LBE). The results showed that uncoated samples developed a pronounced LBE penetration layer after exposure. In contrast, both pack-chromized (PC) coatings and PC + GN composite coatings showed no detectable degradation during the initial exposure.^220^ These coatings acted as effective barriers against LBE corrosion, preventing penetration (schematic mechanism shown in Figure 14C). Similarly, Shen et al.^221^ applied an electrodeposited nickel pretreatment on pure iron substrates, followed by GN (500°C, 5h). The results showed that compared to untreated materials, the wear volume of the dual-treated samples decreased by 68%. However, the ammonia decomposition rate and results shown in Figure 14D indicate that the decomposition rate fluctuated within a similar range for all samples. The presence of the nickel coating seemed to have little effect on ammonia decomposition rate, possibly limited by ammonia flow rate, the complex atmosphere in the reaction chamber, and the relatively low amount of nickel coating in the small samples. The nitrogen generated from ammonia decomposition is related to the ammonia flow rate and tends to stabilize as the flow increases, which is determined by the reaction kinetics on the workpiece surface.

### Gas nitrocarburizing

Gas nitrocarburizing (GNC) is a thermochemical treatment process dominated by nitrogen, in which N and C atoms are simultaneously introduced into the surface layer of a workpiece. This process is also referred to as soft nitriding.^222^ At the processing temperature, nitrogen- and carbon-bearing gases are adsorbed onto the workpiece surface, where they decompose to generate [N] and [C]. Some of these active atoms dissolve into the crystal lattice at the workpiece surface and diffuse inward.^223^ The solubility of carbon in α-Fe is significantly lower than that of nitrogen, causing the near-surface region to become preferentially saturated with carbon. This promotes the precipitation of fine carbides.^224^ These carbides act as nucleation sites, promoting the formation of nitrides and further inward penetration of N atoms. As a result, a compound layer consisting primarily of the ε-phase and γ′-phase forms, followed by a diffusion layer, forms from the surface inward. The ε-phase has a considerable solubility for carbon (up to 3.8 wt. %), which creates favorable conditions for the continued inward diffusion of carbon atoms.^225^ Thus, during GNC, carburizing and nitriding processes mutually enhance each other, acting as diffusion promoters. The carbon source used as the carburizing agent must have a high carburizing potential, low impurity levels, minimal soot formation, and must not react with nitriding agents in a way that reduces the production of [N] and [C] atoms.^226^ The reactions involved in GNC are complex and typically include the following^227^^,^^228^^,^^229^:

(16) $$2{\text{NH}}_{3}⇌N_{2}+3H_{2}$$

(17) $$2{\text{NH}}_{3}⇌2\left[N\right]+3H_{2}$$

(18) $$\text{CO}+H_{2}⇌\left[C\right]+H_{2}O$$

(19) $$2\text{CO}⇌\left[C\right]+{\text{CO}}_{2}$$

(20) $${\text{CO}}_{2}+H_{2}⇌H_{2}O+\text{CO}$$

(21) $$\text{CO}+3H_{2}⇌{\text{CH}}_{4}+H_{2}O$$

(22) $${\text{CH}}_{4}⇌\left[C\right]+2H_{2}$$

**Figure 15:**
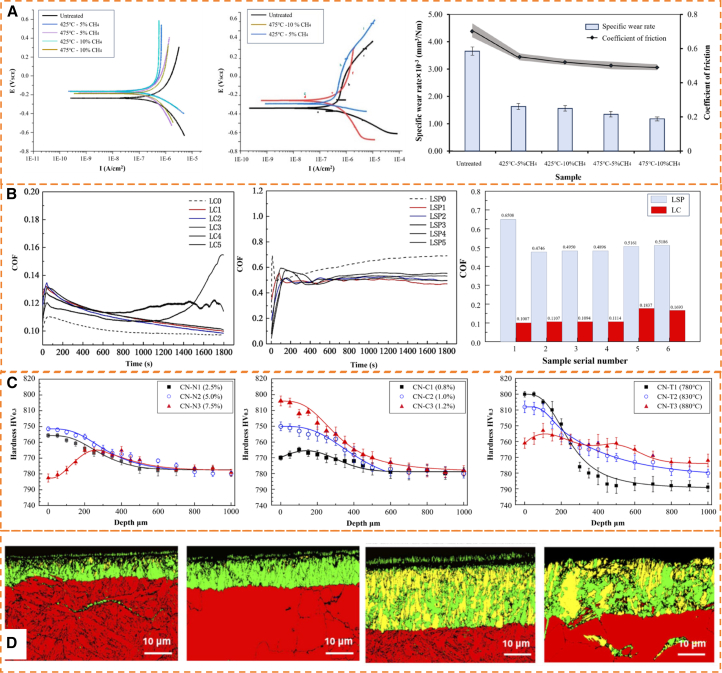
Gas nitrocarburizing of steel (A) Tafel and potential polarization curves of samples in 3.5 wt % NaCl solution under different temperatures and methane contents, specific wear rate, and coefficient of friction of the untreated and treated samples with S-phase layer formed under different temperatures and CH_4_ compositions. (reprinted from Juri et al.^26^ Copyright 2023, with permission from Elsevier). (B) COF values and average COF for LC and LSP treatments, wear tests: 30N, sliding speed: 5 mm/s (reprinted from Ren et al.^235^ Copyright 2021, with permission from Elsevier). (C) Surface hardness profiles of samples under varied processing conditions, tester load:150 kg, dwelling time:5s.^236^ (D) EBSD phase maps of 46MnVS3 and 34CrNiMo6 steels after GN (left) and GNC (right): α-ferrite (red), γ′-phase (green), ε-phase (yellow).^237^

The selection of the carbon source plays a significant role in determining the microstructure, phase composition, and properties of the compound layer formed during GNC.^230^ For example, using methane as the carbon source has been shown to produce a distinct bilayer structure on stainless steel surfaces, consisting of a nitrogen-enriched layer and a carbon-enriched layer.^231^ Studies using C_3_H_8_, CO, CO_2_, CH_4_, and other carbon sources have also resulted in compound layers with varying thicknesses and properties.^232^^,^^233^^,^^234^ Juri et al.^26^ used CH_4_ as the carbon source to study the effects of CH_4_ concentration in the nitriding atmosphere and nitrocarburizing temperature (425°C, 475°C) on the surface properties of 316LVM steel. As shown in Figure 15A, the GNC-treated samples exhibited significantly improved corrosion and wear resistance compared to the untreated samples. The treated GNC samples developed surface phases including γN, γC(111), γC(200), and Fe_2-3_(N, C). The formation of these phases collectively improved the surface properties of the samples. Building on this, Ren et al.^235^ combined LSP with GNC. As shown in Figure 15B, samples treated with this composite process exhibited a COF approximately 20% of that observed with LSP alone. As mentioned earlier, LSP can enhance the diffusion of [N] during GN. This experiment not only validated the theory but also showed that LSP treatment can similarly enhance the diffusion of [C]. This provides a theoretical basis for using hybrid surface treatments to enhance the surface properties of steel in future studies. On the other hand, Wang et al.^236^ studied the effect of GNC on the surface hardness of AISI 52100 steel. The surface hardness profiles under varying treatment conditions are shown in Figure 15C. The results show that introducing a low volume fraction (≤5.0%) of NH_3_ promotes the formation of surface compounds. In contrast, higher ammonia concentrations cause decarburization, leading to a decrease in surface hardness. Similar to GN, increasing both temperature and K_C_ promotes compound formation, thus increasing the average surface hardness of the steel. Yu et al.^237^ applied GN and GNC treatments to 46MnVS3 and 34CrNiMo6 steels. The corresponding EBSD images are presented in Figure 15D. The results show that the GNC-treated samples exhibit a higher volume fraction of the γ′ and ε phases. The ε-phase shows isotropic strain, while the γ′-phase exhibits anisotropic strain. These spatially resolved strain maps, influenced by ε-phase thickness and alloying, reveal new strategies for optimizing wear, fatigue, and corrosion resistance in surface-hardened components.

### Advanced technologies in gas nitriding

While various auxiliary GN techniques significantly improve nitriding efficiency and layer quality, their primary limitation is the need to incorporate additional processes, equipment, or steps. This inevitably increases the overall process cycle time and presents significant challenges in developing novel techniques. To address this efficiency bottleneck, this chapter explores the latest advancements in GN technology. This work systematically reviews the principles and outcomes of advanced technologies (e.g., dynamic controllable nitriding and intelligent monitoring techniques), aiming to establish a strong theoretical foundation and technical resources for developing the next generation of energy-efficient and highly controllable GN processes.

### Low-temperature nitriding

GN is one of the most widely used thermochemical surface treatment methods for steels, extensively applied to improve their fatigue, wear, and corrosion resistance.^238^ Although the GN process has become relatively mature and is widely employed in industrial production, several issues remain to be addressed. For instance, the conventional nitriding temperature is generally above 500°C, and the treatment time often exceeds 30h, resulting in significant energy consumption. For components with stringent dimensional accuracy requirements, the elevated nitriding temperature may lead to distortion, which is unfavorable for the manufacturing of high-precision parts.^13^ In addition, numerous studies have revealed that TGN produces a dense and continuous ε-nitride compound layer (white layer), which provides excellent corrosion resistance. However, this white layer is typically thick and brittle, making it prone to microcracks or even spallation under excessive impact loads or contact stresses.^239^^,^^240^^,^^241^

**Figure 16:**
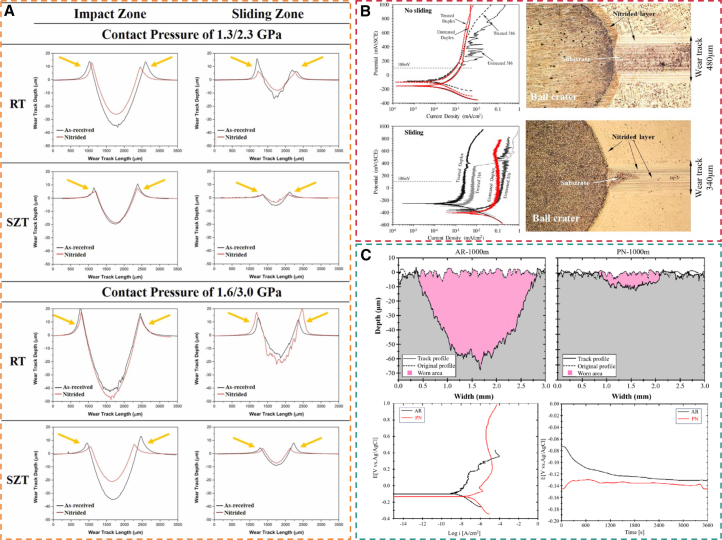
Low-temperature nitriding of steel (A) 2D surface profiles of the impact and sliding zones on wear tracks of AR and nitrided samples tested under RT and SZT conditions (reprinted from Gumuslu et al.^242^ Copyright 2023, with permission from Elsevier) (B) Potentiodynamic polarization curves of nitrided samples under various conditions, along with wear scars of both steels after corrosion testing (reprinted from Haruman et al.^147^ Copyright 2020, with permission from Elsevier). (C) Two-dimensional wear tracks of AR and PN treated samples after 1000 m sliding distance, test load: 20-70N, sliding velocity: 0.1 mm/s. Potential polarization curves in 0.6 M non-deaerated sodium chloride solution (reprinted from Dib et al.^243^ Copyright 2024, with permission from Elsevier).

Low-temperature nitriding (LTN) is a surface modification technique performed at substantially lower temperatures. Studies have shown that combining TGN with RE catalysis, SMAT, or UNSM effectively lowers the nitriding temperature and improves efficiency. However, such hybrid approaches inevitably modify the steel surface topography. Therefore, precise control of the thermal parameters within the TGN process, aimed at achieving low-temperature and high-efficiency nitriding, remains a key direction for future research. In this context, Gumuslu et al.^242^ examined the effect of LTN at 420 °C on the wear resistance of 17Cr-10Ni-2Mo stainless steel at room temperature (RT) and sub-zero temperature (SZT), as shown in Figure 16A. Notably, the nitrided samples exhibited wear rates reduced by 35% at RT and 25% at elevated temperatures compared with the untreated material. Haruman et al.^147^ conducted nitriding treatments on austenitic (316L) and duplex SS (2205). It was demonstrated that LTN significantly enhanced the corrosion resistance of both steels in a 3.5 wt % NaCl solution. The white layer formed at low temperature contributes to reducing both chemical and mechanical wear simultaneously. As illustrated in Figure 16B, the nitrided duplex SS 2205 exhibited superior overall performance compared to the nitrided ASS 316L. Dib et al.^243^ studied the effect of plasma nitriding (PN) at 250 °C on the tribological behavior of super duplex SS. As shown in Figure 16C, the PN-treated sample displayed improved passivation behavior and a markedly lower wear rate than the AR condition.

In summary, although LTN offers advantages in improving the surface characteristics of steels, comprehensive studies systematically exploring this process are still notably scarce in the literature. Notably, LTN is more easily achieved with PN. This is because PN directly ionizes NH_3_ into a glow discharge plasma using a high-voltage direct current electric field within the reaction chamber, resulting in lower temperature dependence.^244^^,^^245^ However, as noted earlier, the high equipment costs associated with PN limit its large-scale industrial application. Consequently, research has focused on optimizing the process parameters of TGN to improve nitrided layer quality while maintaining cost-effectiveness, making it a critical area of focus for overcoming current technological challenges.

### Computer-controlled simulation modeling

Traditionally, the optimization of the TGN process has relied heavily on trial-and-error and empirical knowledge, leading to challenges such as low efficiency, poor consistency, and high resource consumption. However, the integration of intelligent control systems with high-fidelity simulation models is driving transformative breakthroughs in this technology.^246^ The core innovation is the establishment of a multiphysics-coupled simulation model grounded in materials science, thermodynamics, and reaction kinetics. The developed model can faithfully reproduce complex in-furnace phenomena, including gas composition-flow dynamics, heat transfer, chemical reactions, and ultimately, nitrided layer formation.^247^ This capability enables extensive virtual experiments before production, allowing rapid evaluation of how different process parameter combinations affect nitrided layer depth, microstructure morphology, and corrosion and wear resistance. This approach greatly shortens the trial-and-error cycle in nitriding treatments.^248^ However, the prediction accuracy of simulation models strongly depends on reliable boundary conditions and material parameters, highlighting the necessity of intelligent control. In practical furnace operations, advanced sensor networks (e.g., real-time gas analyzers, multi-point temperature sensors, and pressure sensors) enable continuous acquisition of critical in-furnace data, including gas composition, temperature distribution, and pressure.^249^ These real-time data streams are used to calibrate and dynamically update simulation model input parameters, thereby maintaining prediction accuracy under current operating conditions. The closed-loop framework, which combines simulation-based preview optimization with real-time data-driven execution, enables global process optimization.^250^ This approach shortens process development cycles, lowers trial-and-error costs, and ensures high consistency and reproducibility in mass production.^251^^,^^252^ At the same time, the integrated knowledge from simulation models and operational data evolves iteratively, creating a robust process knowledge base. This knowledge base supports rapid process development for new workpieces and materials.^253^

**Figure 17:**
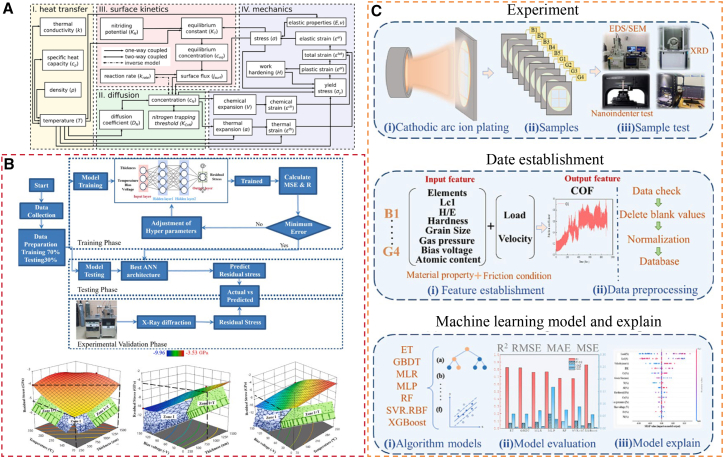
Computer-controlled simulation modeling (A) Schematic of the numerical methodology for the finite difference model (reprinted from Kücükyildiz et al.^254^ Copyright 2020, with permission from Elsevier) (B) Integrated prediction framework for residual stresses and their evolution in TiN-sputtered coatings.^255^ (C) Process flowchart for fabricating high-entropy nitride coatings and predicting COF via machine learning (reprinted from Jia et al.^256^ Copyright 2024, with permission from Elsevier).

In recent years, researchers have applied integrated intelligent control and simulation modeling to GN. These methods enable real-time monitoring of parameter variations during the nitriding process and allow the prediction of the surface properties of nitrided samples. Kücükyildiz et al.^254^ developed a multiphysics-coupled implicit finite difference model. As shown in Figure 17A, this model simulates the temporal evolution of surface composition and RCS during LTN of steel. The one-dimensional model incorporates multiple mechanisms along the depth direction, including nitrogen diffusion, elastic-plastic deformation from lattice expansion, stress-gradient-driven nitrogen diffusion, solid-solution strengthening by nitrogen, and chromium trapping of nitrogen. The model was calibrated using surface reaction kinetics parameters as fitting variables, constrained by experimentally measured mass absorption curves. The model predictions showed excellent agreement with experimental results. On the other hand, Abdullah et al.^255^ employed a feedforward artificial neural network (ANN) to predict the average RCS in TiN coatings with varying thicknesses. The ANN model was validated by comparing its predictions with experimental data, showing good agreement. Atomic force microscopy (AFM) analysis further revealed the growth mechanism underlying the evolution of residual stresses, as illustrated in Figure 17B. Jia et al.^256^ fabricated nine distinct high-entropy alloy coatings. Mechanical testing showed that the COF increased with increasing applied load. Using 53 datasets for machine learning (ML), seven regression models were developed to predict the COF from input features, including mechanical properties such as nanohardness. Figure 17C presents the process flowchart for fabricating high-entropy nitride coatings and predicting results using ML. The results demonstrate that critical load, applied load, chromium content, and gas pressure have the greatest influence on the COF, thereby validating the model’s effectiveness in predicting COF for high-entropy coatings.

As a key process for enhancing material surface properties, GN is undergoing a major transformation. Its traditional reliance on empirical parameter adjustment is being replaced by intelligent control and high-precision simulation modeling techniques. An intelligent control system, supported by a real-time sensor network, dynamically regulates ammonia flow rate, decomposition rate, and temperature profiles through adaptive algorithms, thereby reducing the impact of furnace-load variations and process fluctuations.^257^ The finite difference model accurately predicts the evolution of surface composition gradients and residual stresses.^258^ Such models accelerate the development of new nitriding processes by more than 50%, substituting costly trial-and-error methods with “virtual experiments.” ML models achieve high-precision prediction of key properties, such as the COF, by integrating process parameters, microstructure, and mechanical properties. This provides a quantitative basis for coating design.^259^ In summary, intelligent technologies and simulation modeling have greatly improved the efficiency and consistency of GN, laying a critical foundation for the digital transformation of surface-enhancement technologies in high-end equipment.

### Comparison of different nitriding technologies

GN has a long history, and a variety of assisted GN methods have now been developed. Each method has its own advantages and is applied in different fields. Table 5 systematically summarizes recent research findings on GN technology applied to various steel substrates. Research shows that precise control of nitriding temperature, duration, atmosphere composition, and auxiliary methods allows the optimization of the compound layer’s thickness, density, and phase constitution. This results in a modified surface layer characterized by high hardness, low brittleness, and excellent corrosion resistance. These findings not only clarify the performance limits of current GN technology in improving steel performance, but also provide a solid theoretical foundation and clear directions for process optimization.

**Table 5:** Summary of performance characteristics of various catalytic GN technologies

Process	Key parameters	Performance improvement	Application
UNSM + GN	Temperature: 500°C–600°C; Time: 2–10 h; Gas composition: NH_3_ (ammonia) or N_2_+H_2_ mixture; Pressure: atmospheric or low pressure; Nanostructuring method: mechanical grinding or ultrasonic impact.	Surface hardness increase (up to 1000–1500 HV); enhanced wear resistance; improved fatigue strength; increased diffusion layer depth (10–50 μm).	Tool steels, molds, bearings, and other components require high wear resistance and fatigue strength.
SP + GN	Shot peening parameters: shot size (0.1–0.5 mm), intensity (0.2–0.6 mmA); Nitriding temperature: 500°C–580°C; Time: 2–8 h; Gas composition: NH_3_.	Surface hardness increase (900–1300 HV); improved wear and corrosion resistance; residual compressive stress layer depth up to 100–200 μm.	Aerospace engine components, automotive gears, crankshafts, and other dynamically loaded parts.
SMAT + GN	Mechanical treatment parameters: load (50–200 N), speed (100–500 rpm); Nitriding temperature: 500°C–600°C; Time: 2–6 h; Gas composition: NH_3_.	Substantial hardness increase (1000–1600 HV); enhanced wear and corrosion resistance; more uniform diffusion layer (depth 20–60 μm).	Precision tools, cutting tools, and high-performance molds.
LSP + GN	Laser parameters: power (500–2000 W), scanning speed (10–100 mm/s), spot size; Nitriding temperature: 500-580C; Time: 1–5 h; Gas composition: NH_3_.	Extremely high local hardness (1200–1800 HV); improved wear and heat resistance; controllable diffusion layer depth (10–40 μm).	Tool and mold localized strengthening, medical devices, and aerospace high-temperature components.
POGN	Pretreatment oxidation temperature: 300°C–500°C; Time: 0.5–2 h; Nitriding temperature: 500°C–570°C; Time: 2–8 h; Gas composition: NH_3_ or N_2_-H_2_ mixture.	More uniform diffusion layer; hardness increase (800–1200 HV); enhanced corrosion and oxidation resistance; diffusion layer depth 20–50 μm.	Stainless steel, heat-resistant steels, and chemical equipment parts.
RE catalyzed GN	Rare earth types: CeO_2_, La_2_O_3_; Concentration: 0.1–1 wt %; Nitriding temperature: 500°C–580°C; Time: 2–6 h; Gas composition: NH_3_ + rare earth compounds.	Increased nitriding rate by 20–50%; hardness increase (1000–1400 HV); deeper diffusion layer (30–70 μm); improved wear and fatigue resistance.	High-efficiency nitriding, automotive engine parts, heavy machinery components.
GNC	Temperature: 570°C–590°C; Time: 1–4 h; Gas composition: NH_3_ + carburizing gases (e.g., CO and CH_4_); Pressure: atmospheric.	High surface hardness (800–1100 HV); excellent wear and fatigue resistance; some toughness; diffusion layer depth 10–30 μm.	Gears, bearings, shaft parts, and structural components.
Alloying elements catalyzed GN	Alloying elements: Cr, Al, Ti (content 1–5%); Nitriding temperature: 500°C–600°C; Time: 2–8 h; Gas composition: NH_3_.	Significant hardness increase (1000–1500 HV); enhanced heat and corrosion resistance; diffusion layer depth 20–60 μm.	Alloy steels, titanium alloys, and aerospace high-temperature components.
LTN	Temperature: 400°C–500°C; time: 4–12 h; gas composition: NH_3_ or N_2_-H_2_ mixture; pressure: low or atmospheric.	Moderate hardness (600–1000 HV); good dimensional stability; improved wear resistance; shallower diffusion layer (5–20 μm).	Precision instruments, electronic components, thin-wall parts, stainless steel products.
Simulation modeling	Model types: diffusion model, phase-field model; Input parameters: temperature, time, gas composition, material properties; Outputs: diffusion layer depth, hardness distribution.	Optimizes nitriding uniformity and efficiency; predicts performance improvement (e.g., hardness and residual stress); reduces experimental costs.	Process development, quality control, new material research, and industrial production optimization.

## Summary and perspectives

This article provides a comprehensive review of the fundamental mechanisms and microstructural evolution associated with GN. The discussion encompasses the complete sequence of gas-phase reactions, surface adsorption, and substrate diffusion, with particular emphasis on how processing conditions govern the formation and evolution of the ε/γ′ compound layers and the diffusion layer.

The findings indicate that the efficiency of active nitrogen formation, the strength of surface adsorption, and the diffusion behavior within the lattice are key factors governing the thickness, phase composition, and overall properties of the nitrided layer. Adjusting temperature, K_N_, gas composition, and surface condition can significantly affect the density of the compound layer, the nitrogen concentration gradient in the diffusion layer, and the residual stress distribution. The microstructure of the nitrided layer directly determines the wear and corrosion resistance of steel. A dense and uniform ε/γ′ compound layer provides high hardness and strong resistance to adhesive wear, whereas a thick diffusion layer and its induced compressive stresses enhance resistance to fatigue wear and microcrack propagation. High-nitrogen solid solutions, nanoscale precipitates, and interface strengthening within the nitrided layer further improve long-term stability under coupled corrosion-wear conditions. However, issues such as heterogeneous phases, porous compound layers, and insufficient ammonia dissociation may still lead to localized corrosion, spalling, or accelerated wear.

Surface nanocrystallization (UNSM, SMAT, SP) and laser- or impact-based treatments can increase grain boundary density and compressive stress at the surface while reducing the activation energy for nitrogen diffusion. These effects shorten the nitriding cycle and improve layer density and fatigue resistance. Pre-oxidation, rare-earth or alloy-element catalysis, and pre-deposited metallic coatings can accelerate the formation and diffusion of active nitrogen by promoting NH_3_ dissociation or enabling additional diffusion pathways. However, careful control is required to avoid the formation of brittle phases or interfacial defects. Gas nitrocarburizing provides superior surface hardness and fatigue performance but requires tighter control of temperature and gas composition. Low-temperature or low-pressure processes, together with computer-controlled monitoring, offer advantages in maintaining dimensional accuracy while reducing energy consumption and emissions.

Overall, existing studies demonstrate that GN enhances both wear and corrosion resistance by modifying surface chemistry, stress state, and microstructure. However, achieving a controllable and efficient nitriding process suitable for complex service conditions requires further advances in mechanistic understanding and process optimization. Future research should continue to progress along the following directions.

### Advance mechanistic understanding of active nitrogen generation and diffusion

Current models describe ammonia decomposition, surface adsorption, and lattice diffusion primarily in macroscopic terms. Future work should integrate *in-situ* characterization, first-principles calculations, and multiscale simulations to elucidate the migration pathways of active nitrogen in the gas phase, at interfaces, and within the bulk. These insights will enable more accurate microscale nitriding models and support controlled growth of both the compound and diffusion layers.

### Establish synergistic strengthening mechanisms for improving wear and corrosion resistance

Coupled wear-corrosion remains the dominant failure mode of nitrided layers under complex service conditions. Future efforts should focus on reducing porosity in the compound layer, tailoring the ε/γ′ phase ratio, stabilizing interfacial bonding, and introducing nanoscale precipitates and beneficial compressive stresses. These strategies can facilitate the development of integrated resistance mechanisms capable of mitigating adhesive wear, fatigue wear, and localized corrosion simultaneously.

### Develop hybrid techniques to promote nitrogen adsorption and diffusion

Techniques such as surface nanocrystallization, laser- or impact-based strengthening, pre-oxidation, rare-earth catalysis, and metallic coatings show considerable potential for accelerating nitrogen adsorption and diffusion. However, their interactions and long-term stability remain insufficiently understood. Future efforts should investigate the coupling effects of combined treatments and establish synergistic pathways that enhance nitrogen diffusion, enabling denser, more uniform, and more stable nitrided layers.

## Acknowledgments

This work was supported by the 10.13039/501100001809National Natural Science Foundation of China (Grant No. 22362005), Ph.D. Programs Foundation of 10.13039/501100015920Guangxi University of Science and Technology (Grant No. 23Z04).

## Author contributions

J.L.: resources, investigation, and funding acquisition. H.S.: writing – review and editing, writing – original draft, methodology, and investigation. K.L.: supervision, project administration, and conceptualization.

## Declaration of interests

The authors declare no conflicts of interest for this work.
